# Rehabilitation of Frozen Shoulder: A Systematic Review and Meta‐Analysis of Multifactorial Interventions

**DOI:** 10.1155/prm/3820129

**Published:** 2026-06-17

**Authors:** Dina Hamed-Hamed, Jose Javier Pérez Montilla, Fabrizio Brindisino, Filip Struyf, Santiago Navarro-Ledesma

**Affiliations:** ^1^ Clinical Medicine and Public Health PhD Program, Faculty of Health Sciences, University of Granada, Granada, Spain, ugr.es; ^2^ Hospital Universitario de Melilla, C. Luis de Ostáriz 12, Melilla, Spain; ^3^ Department of Physical Therapy, Faculty of Health Sciences, Campus of Melilla, University of Granada, Querol Street 5, Melilla, Spain, ugr.es; ^4^ Department of Medicine and Health Science “Vincenzo Tiberio”, University of Molise, Campobasso, Italy, unimol.it; ^5^ Research Group MOVANT, Department of Rehabilitation Sciences and Physiotherapy, Faculty of Health Sciences, University of Antwerp, Antwerp, Belgium, uantwerpen.be

**Keywords:** frozen shoulder, healthy lifestyle, metabolism, pain, psychological factors

## Abstract

**Introduction:**

Frozen shoulder (FS) is characterized by a multifactorial progression, often worsened by metabolic factors such as diabetes. Effective management may require a multidisciplinary approach that includes therapeutic exercise, physiotherapy, metabolic control, psychological support, and healthy lifestyle interventions. However, current evidence remains fragmented and predominantly focused on isolated domains, with limited integration of these factors within a biopsychosocial framework. The aim of this study was to comprehensively evaluate the influence of exercise‐based interventions, as well as metabolic, psychological, and lifestyle‐related factors, on clinical outcomes, including pain, function, range of motion, and quality of life, in patients with FS.

**Methods:**

This systematic review with meta‐analysis, reported following the PRISMA guidelines, included 31 studies published between 2010 and 2024, comprising randomized controlled trials, observational studies, Mendelian randomization studies, and qualitative studies. MEDLINE, Web of Science, CINAHL, SPORTDiscus, and Scopus were searched until April 2025. The risk of bias was assessed according to study design. The Cochrane Risk of Bias tool (RoB 1) was used for randomized controlled trials. Observational studies (including cross‐sectional, case–control, and cohort studies) were assessed using the Newcastle–Ottawa scale (NOS). Mendelian randomization studies were evaluated using established methodological quality criteria for MR studies, including the assessment of instrument validity, pleiotropy, and heterogeneity. Qualitative studies were appraised using appropriate qualitative assessment criteria. The certainty of evidence was rated using the Grading of Recommendations, Assessment, Development and Evaluation (GRADE) approach.

**Results:**

Twelve studies evaluated physical interventions. Global effect summary of exercise versus control showed a small but statistically significant benefit favoring exercise (standardized mean difference [SMD], Hedges’ *g* = 0.10; 95% CI 0.05–0.15) across clinical outcomes, particularly pain, function, and range of motion. Associations between FS and metabolic parameters (e.g., triglycerides, glucose, and HbA1c) were identified in 12 studies, although these results were synthesized narratively and no pooled effect estimates (e.g., odds ratios) were calculated due to heterogeneity. Seven studies showed that anxiety was consistently associated with FS severity, with potential impact on pain, functional outcomes, and quality of life, although this relationship may depend on sample characteristics. The risk of bias was low in exercise‐related studies, unclear in those on quality of life, and high in metabolic studies. Overall, the certainty of evidence was rated as very low due to the risk of bias, heterogeneity, and imprecision.

**Conclusions:**

Evidence supports a multifactorial, biopsychosocial model for FS. Exercise‐based interventions were associated with small improvements in pain, function, and range of motion, while metabolic dysfunction and anxiety were identified as factors associated with FS severity. However, the certainty of evidence was very low and findings should be interpreted with caution due to high heterogeneity and methodological variability across studies. Future research should prioritize integrated, multidisciplinary strategies to improve FS treatment.

## 1. Introduction

Frozen shoulder (FS), also known as adhesive capsulitis, is a musculoskeletal disorder characterized by pain, stiffness, and progressive loss of movement in the glenohumeral joint [1]. It affects approximately 2%–5% of the general population, with higher incidence in women aged 40 and 60 years [1, 2]. FS can be classified as primary (idiopathic) or secondary, the latter being associated with trauma, prior surgery, endocrine disorders, or metabolic alterations—most notably diabetes mellitus [3, 4].

The association between FS and metabolic dysfunctions is well documented [5]. In individuals with Type 1 diabetes, the incidence of FS ranges from 10% to 20% [6, 7], while in Type 2 diabetes, prevalence estimates vary between 13% and 30%, depending on glycemic control and disease duration [8, 9]. Additionally, metabolic syndrome, which includes visceral obesity, dyslipidemia, hypertension, and insulin resistance, creates a proinflammatory environment that contributes to both the development and severity of FS [10–12].

From a pathophysiological perspective, FS is related to chronic inflammatory processes mediated by cytokines and immunometabolic mechanisms that result in capsular fibrosis [13–15]. These processes may also affect the central nervous system, contributing to psychological symptoms such as low mood, catastrophizing, anxiety, and depression, which in turn are associated with increased pain perception and poorer functional recovery [16–18]. Despite the substantial clinical and economic burden, research on the psychological and social implications of FS—as well as patients’ perspectives and needs—remains limited. However, emerging studies have begun to emphasize the perspectives, perceptions, and expectations of patients with FS, highlighting the need for more holistic and patient‐centered treatment approaches [19].

In fact, the impact of FS on the quality of life is considerable [20, 21]. Persistent pain and functional limitations compromise patients’ autonomy and often lead to sleep disturbances, especially due to nocturnal pain [7]. Sleep disruption can further hinder tissue repair, exacerbate pain sensitivity, and perpetuate inflammation [22]. However, further studies are needed to integrate sleep assessment and management in the treatment of patients with chronic musculoskeletal pain [23].

Current management of FS typically involves a combination of conservative and, in selected cases, invasive approaches. Physiotherapy and exercise‐based interventions are considered the cornerstone of treatment, with the primary goals of restoring range of motion (ROM), reducing pain, and improving function [24]. Rehabilitation strategies that combine manual therapy, progressive exercise, and patient education have shown clinically meaningful improvements and are widely recommended in recent clinical literature [25]. Moreover, contemporary approaches increasingly recognize the importance of integrating psychosocial and behavioral components into physiotherapy, supporting a shift toward more patient‐centered and biopsychosocial models of care [26].

Pharmacological interventions, such as nonsteroidal anti‐inflammatory drugs and intra‐articular corticosteroid injections, are frequently used to manage pain and inflammation, particularly in early stages, where they have demonstrated short‐term improvements in pain and function [27, 28]. In addition, hydrodilatation has been reported as an effective adjunct treatment, showing improvements in pain, disability, and ROM, especially in the short term [29]. Emerging minimally invasive approaches, including suprascapular nerve blocks and ultrasound‐guided interventions, have also gained increasing attention for their ability to reduce pain and facilitate engagement in rehabilitation programs [30, 31]. These neuromodulatory techniques, sometimes considered within the broader concept of neurolysis or peripheral nerve modulation, may enhance treatment outcomes when combined with exercise‐based therapy, although current evidence remains limited and heterogeneous [32].

However, despite the availability of multiple treatment options, FS should not be approached as a purely mechanical disorder. Recent contributions from leading researchers in the field have emphasized the importance of consistent terminology, individualized treatment, and the integration of biological, psychological, and contextual factors in FS management. This evolving perspective supports the adoption of a biopsychosocial framework in both research and clinical practice.

Despite growing evidence linking FS with metabolic, psychological, and lifestyle‐related factors, current research remains largely fragmented and predominantly focused on isolated domains, particularly biomechanical and rehabilitation approaches. Consequently, there is a lack of integrated evidence evaluating FS within a comprehensive biopsychosocial framework [33, 34].

While physiotherapy and therapeutic exercise remain essential components due to their effects on mobility and pain modulation, adjunctive strategies may also be beneficial [15, 35]. These include metabolic control (e.g., dietary modifications, and regulation of glucose and lipid levels), psychological interventions, and sleep optimization strategies (such as cognitive‐behavioral therapy, meditation, or sleep hygiene) [7, 36–39].

Multifactorial interventions, which combine exercise‐based therapy with metabolic, psychological, and lifestyle‐related strategies, have been proposed as a promising approach for the management of FS [32]. These interventions may provide synergistic effects by simultaneously targeting pain, inflammation, functional limitation, and psychosocial factors, potentially leading to improved clinical outcomes and patient satisfaction [25, 40]. However, despite their theoretical advantages, multifactorial approaches also present important limitations. These include increased complexity in intervention design, difficulties in standardization and reproducibility, and challenges in identifying the relative contribution of each component to the overall effect. Additionally, variability in patient characteristics and adherence may influence treatment response, limiting the generalizability of findings across populations [25, 41].

Therefore, a comprehensive synthesis of the available evidence addressing these multidimensional factors is needed to better understand their role in FS symptomatology and management.

From a clinical perspective, this review provides relevant insights for different stakeholders. For clinicians, it supports the integration of exercise‐based therapy with metabolic and psychological considerations, facilitating more comprehensive and individualized treatment strategies. For researchers, it highlights current gaps in the literature, particularly the need for well‐designed studies evaluating multifactorial interventions and standardized outcome measures. For patients, this approach emphasizes the importance of addressing not only physical symptoms but also broader aspects such as metabolic health, psychological well‐being, and lifestyle habits, which may influence recovery and overall quality of life. Therefore, this review contributes to bridging the gap between evidence and clinical practice, supporting a more holistic and patient‐centered management of FS.

In this context, the present systematic review and meta‐analysis aim to comprehensively evaluate the influence of exercise‐based interventions, as well as metabolic, psychological, and lifestyle‐related factors, on pain, function, and overall clinical outcomes in patients with FS.

## 2. Methods

### 2.1. Study Design

A systematic review and meta‐analysis were reported following the guidelines established by the Preferred Reporting Items for Systematic Review and Meta‐Analysis (PRISMA) [42] standard, incorporating experimental studies. The Population, Intervention, Comparison, Outcome, and Study (PICOS) design strategy was applied for the development of search strings. Additionally, the review protocol was prospectively registered in the International Prospective Register of Systematic Reviews (PROSPERO; Registration Number: CRD42025645064).

The review included studies published between 2010 and 2024.

The objective of this systematic review was to gather scientific evidence on the influence of physical activity, metabolic and psychological profiles, as well as lifestyle habits, on the pain experienced by patients with FS.

### 2.2. Documentary Sources Consulted

The following electronic databases were reviewed: MEDLINE, Web of Science, CINAHL, SPORTDiscus, and Scopus. To broaden our search, we also investigated study registers such as ClinicalTrials.gov and other gray literature, including Google Scholar and conference databases. To identify all potentially relevant studies, we conducted a manual cross‐reference of the reference lists from the included studies.

The search was conducted without restriction on geographic setting, including studies from different countries and clinical contexts. The last search was performed on April 21, 2025.

### 2.3. Search Strategy

To design the search strategy, keywords were selected using both controlled vocabulary and free‐text terms. Medical Subject Headings (MeSH) terms included the following: “Fasting,” “Caloric Restriction,” “Diet, Ketogenic,” “Physical Therapy Modalities,” “Rehabilitation,” “Exercise,” “Pain,” “Chronic Pain,” “Healthy Lifestyle,” “Quality of Life,” “Circadian Clocks,” “Circadian Rhythm,” and “Sleep Wake Disorders.” Additionally, free‐text (non‐MeSH) terms were incorporated, such as “frozen shoulder,” “adhesive capsulitis,” “nutritional strategies,” “physiotherapy,” “physical therapy,” “healthy,” “quality of life,” “psychological factors,” and “functionality.” These terms were combined using the logical operators AND OR, ensuring their presence in the title, abstract, and keywords.

The search strategy was structured into concept blocks, including (1) the condition (FS/adhesive capsulitis) and (2) multifactorial domains relevant to the research question (exercise/rehabilitation interventions and metabolic, psychological, and lifestyle‐related exposures). This approach was adopted to comprehensively capture the existing scientific evidence addressing the multifactorial nature of FS, particularly in relation to lifestyle factors such as nutrition, metabolism, and physical activity.

To optimize the search, the following filters were applied: peer‐reviewed publications in English, published within the last 15 years.

The search process, as well as the article selection and the risk of bias assessment, was conducted by two authors. The last search was conducted on April 21, 2025.

The search strategy was intentionally designed to maximize sensitivity by including a broad range of lifestyle‐related terms. Although some terms (e.g., circadian rhythm, fasting, dietary strategies) may reduce specificity and retrieve nonrelevant records, strict screening and eligibility criteria were applied to ensure that only studies directly addressing FS and the predefined domains were included. Minor syntax adjustments were applied to ensure database compatibility without altering the conceptual structure of the search strategy.

Table 1 outlines the search strategy used in MEDLINE (accessible through PubMed), while supporting information contains the remaining search strategies (Supporting Information A).

**TABLE 1:** MEDLINE search strategy.

Database	Search strategy
MEDLINE (PubMed)	(“Frozen shoulder”) AND ((“Circadian Clocks”[Mesh] OR “Circadian Rhythm”[Mesh] OR “Chronobiology “Disorders”[Mesh] OR “Chronobiology Discipline”[Mesh] OR “Sleep Wake Disorders”[Mesh] OR “insulin”[Mesh] OR “Fasting”[Mesh] OR “Caloric Restriction”[Mesh] OR “Diet, Ketogenic”[Mesh]) AND (“Pain”[Mesh] OR “Chronic Pain”[Mesh] OR “Healthy Lifestyle”[Mesh] OR “Quality of Life/psychology”[Mesh])) OR ((“Adhesive capsulitis” [tw] OR “Frozen shoulder” [tw]) AND (“Circadian Clocks”[tw] OR “Circadian Rhythm”[tw] OR “Chronobiology “Disorders”[tw] OR “Chronobiology Discipline”[tw] OR “Sleep Wake Disorders”[tw] OR “insulin”[tw] OR “Fasting”[tw] OR “Caloric Restriction”[tw] OR “Diet, Ketogenic”[tw] OR “Nutritional strategies” [tw] OR “therapy” [tw]) AND (“Pain” [tw] OR “Chronic Pain”[tw] OR “Healthy”[tw] OR “Healthy Lifestyle”[tw] OR “Quality of life” [tw] OR “Quality of Life/psychology”[tw] OR “Psychological factors” [tw] OR “Functionality” [tw]))

### 2.4. Selection Criteria

The selection process was conducted by two reviewers.

• Inclusion criteria
 All observational and experimental studies that investigated the influence of physical exercise strategies, metabolic and psychological profiles, and lifestyle habits on the symptomatology of patients with FS were included.
• Exclusion criteria
 Studies were excluded if participants had previously undergone surgery or were on a surgical waiting list (i.e., with a confirmed surgical indication).
 Studies in which exercise was administered solely as part of a postcorticosteroid injection protocol, or in immediate combination with corticosteroid injections, were also excluded, as it was not possible to isolate the effect of exercise from other medical interventions.

### 2.5. Study Selection Process

The Rayyan QCRI program [43] was used to remove duplicate studies identified in the different databases. After removing the duplicates, two reviewers independently (D.H‐H. and S.N‐L.) screened titles and abstracts according to the predefined inclusion and exclusion criteria. Subsequently, full‐text articles of potentially eligible studies were assessed for inclusion.

The level of agreement between reviewers was assessed through independent screening, and any discrepancies were resolved through discussion and consensus with a third reviewer (F.B.). Due to the consensus‐based resolution process, a formal kappa statistic was not calculated.

One reviewer (D.H‐H.) was responsible for overseeing the decisions made by the other two.

### 2.6. Outcome Measures

The primary outcomes of this review were pain, physical function, and ROM in patients with FS. Pain was commonly assessed using validated instruments such as the visual analog scale (VAS) or numeric rating scale (NRS). Physical function and disability were primarily evaluated using patient‐reported outcome measures (PROMs), including the Shoulder Pain and Disability Index (SPADI), the Disabilities of the Arm, Shoulder and Hand (DASH) questionnaire, and the Constant–Murley score. ROM was assessed using goniometric measurements.

These outcome measures are widely used in clinical and research settings and have demonstrated good reliability and validity in patients with shoulder disorders, supporting their use for assessing treatment effectiveness and clinical progression.

### 2.7. Protocol Amendments and Outcome Selection

The review protocol was prospectively registered in PROSPERO (No. CRD42025645064). During the review process, no major deviations from the original protocol were made. However, some planned outcomes could not be quantitatively synthesized due to substantial heterogeneity in study design, interventions, outcome measures, and reporting methods.

Specifically, while multiple domains were initially considered (including metabolic, psychological, and lifestyle‐related outcomes), only exercise‐based interventions provided sufficient homogeneous data to allow quantitative pooling in the meta‐analysis. Other outcomes were therefore synthesized narratively.

These decisions were made in accordance with PRISMA recommendations, ensuring transparency and methodological rigor, and to avoid inappropriate pooling of heterogeneous data that could compromise the validity of the findings.

### 2.8. Data Extraction

Two reviewers independently extracted data using a standardized form. To enhance reproducibility, we extracted intervention and exposure characteristics using a structured template. For exercise/rehabilitation studies, we recorded modality (e.g., mobilization, stretching, neuromuscular exercise, proprioceptive neuromuscular facilitation [PNF]), dosage (frequency, session duration, total duration), intensity and progression criteria, supervision (supervised vs. home‐based), cointerventions, and adherence strategies (when reported). For psychological factors/interventions, we extracted the construct assessed (e.g., anxiety, depression, catastrophizing), the instrument used, timing of assessment, and whether any psychological support/education component was delivered. For lifestyle‐related factors, we extracted any reported sleep‐related variables, physical activity level measures, dietary patterns or other lifestyle exposures, and their measurement instruments. When key parameters were not reported in the primary study, we coded them as “not reported.” The following information was collected: study characteristics (author, year of publication, study design, and location), sample characteristics (sample size, age, and gender), intervention characteristics (type of intervention), variables of interest (physical exercise, metabolic and psychological profile, anxiety, and lifestyle habits), and main outcomes (assessment tools, follow‐up duration, and intervention results). All data were recorded in an Excel spreadsheet. One researcher performed the initial data extraction, which was independently verified by a second reviewer.

In cases where data of interest were missing or incomplete, the corresponding authors were contacted by email. Following five unsuccessful contact attempts or a confirmation from the authors that the data were lost, the data were designated as “missing.” Conversely, if the authors provided the required data, it was incorporated by the reviewers in the same Excel spreadsheet.

### 2.9. Risk of Bias Assessment Tool

The risk of bias of the included studies was assessed according to study design using appropriate, design‐specific tools.

Randomized controlled trials (RCTs), including the pilot randomized controlled study, were assessed using the Cochrane Risk of Bias tool (RoB 1), which evaluates domains such as selection bias, performance bias, detection bias, attrition bias, reporting bias, and other sources of bias. Each domain was classified as “low risk,” “high risk,” or “unclear risk.”

Observational studies (including cohort, case–control, and cross‐sectional designs) were assessed using the Newcastle–Ottawa scale (NOS), which evaluates selection, comparability, and outcome/exposure domains.

Mendelian randomization studies were not assessed using conventional risk‐of‐bias tools; instead, they were appraised qualitatively based on key methodological assumptions (relevance, independence, and exclusion restriction) and synthesized narratively.

Qualitative studies were assessed using the Critical Appraisal Skills Programme (CASP) checklist.

The risk of bias was independently evaluated by two reviewers. Any disagreement over the assessment was resolved through discussion with a third author (F.B).

### 2.10. Quality of the Evidence

The Grading of Recommendations, Assessments, Development, and Evaluation (GRADE) system [44] was employed to evaluate the quality of evidence for the reported outcomes in the studies. The GRADE approach is a system for rating the quality of a body of evidence in systematic reviews and offers a structured process for developing and presenting evidence summaries. As often systematic reviews provide a comprehensive summary of the evidence without typically not including healthcare recommendations, the use of the GRADE approach terminates after rating the quality of evidence for outcomes investigated and clearly presenting the results through a transparent process of moving from evidence to clinical use. Factors considered in the quality assessment include risk of bias in the study, inconsistency, imprecision, publication bias, indirect results, and other elements that could influence evidence quality. Two reviewers independently evaluated the quality of evidence and disagreements were resolved by discussion with a third author (XY). The classification of evidence quality was outlined as follows: (1) high (future studies are unlikely to significantly change our confidence in the effect estimate, and there is no known or suspected reporting bias: all areas are met); (2) moderate (future research is expected to have a considerable impact on our confidence in the effect estimate and could alter it: one area is unmet); (3) low (future studies are likely to significantly affect our confidence in the effect estimate and may change it: two areas are unmet); and (4) very low (our confidence in the estimate is uncertain: three areas are unmet).

The GRADEpro GDT was used to generate the GRADE Summary (GRADEpro, 2023) [45]. In addition, we present the GRADE assessment in a structured summary of findings table in the main manuscript for the key patient‐centered outcomes. The complete GRADE assessment is provided in the Supporting Information (Table C).

### 2.11. Intervention Definition

To address potential inconsistencies in terminology across studies, interventions were categorized using standardized definitions.

Exercise‐based interventions were defined as structured therapeutic programs including active or assisted range‐of‐motion exercises, strengthening exercises, neuromuscular training, or functional task‐oriented exercises, delivered in either supervised or home‐based formats.

Physiotherapy or conventional care was defined as interventions primarily based on passive modalities (e.g., manual therapy, electrotherapy, or general advice), with or without adjunct exercise components.

When studies included mixed or multimodal interventions, classification was based on the predominant therapeutic component.

### 2.12. Overlapping Analysis

Based on the included studies, two types of analyses were conducted:

Quantitative synthesis (meta‐analysis) was conducted exclusively for controlled intervention studies comparing exercise/rehabilitation versus a control or comparator condition. All other study designs, including observational, Mendelian randomization, and qualitative studies, were synthesized narratively and were not included in the quantitative pooling of results.

#### 2.12.1. Meta‐Analysis of Exercise Interventions

For studies comparing exercise/rehabilitation versus a control/comparator condition, we quantified the intervention effect using the standardized mean difference (SMD; Hedges’ g), as included studies reported patient‐centered outcomes using different instruments and scales.

MDs were not used due to the heterogeneity of outcome measures across studies.

The effect estimate was based on between‐group differences in pre–post change (ΔExercise − ΔControl), where Δ represents the change from baseline to postintervention within each arm. When change‐score standard deviations (SDs) were not reported, we used the corresponding reported dispersion measures following standard meta‐analytic guidance and software defaults.

Effect sizes were pooled using a random‐effects inverse‐variance model given the expected clinical and methodological heterogeneity (e.g., differences in exercise protocols, intervention dose, supervision, population characteristics, and outcome measures).

Effects were oriented so that positive values reflected improvement in favor of exercise. Statistical heterogeneity was assessed using Cochran’s Q and the *I* ^2^ statistic, with *I* ^2^ values of approximately 25%, 50%, and 75% interpreted as low, moderate, and high heterogeneity, respectively. When heterogeneity was substantial, pooled estimates were interpreted cautiously as an average effect across heterogeneous settings rather than as a single universally applicable estimate [46, 47].

The magnitude of effect sizes was interpreted according to conventional thresholds for SMDs, where values of approximately 0.2 were considered small, 0.5 moderate, and 0.8 large. Clinical relevance was interpreted cautiously, taking into account both the magnitude and consistency of effects across studies, given the heterogeneity of interventions and outcome measures. Minimal clinically important differences (MCIDs) were not predefined due to variability in outcome instruments across studies.

When SDs of change scores were not reported, they were estimated using standard methods recommended in the Cochrane Handbook. Specifically, SDs were derived from available baseline and postintervention SDs, assuming a correlation coefficient between repeated measurements. In the absence of reported correlations, a conservative correlation coefficient was assumed in line with common meta‐analytic practice. Given the limited reporting in the original studies, sensitivity analyses using alternative correlation coefficients were not performed, and this should be considered when interpreting the results.

### 2.13. Subgroup Analysis and Meta‐Regression for Heterogeneity Assessment

Subgroup analyses and meta‐regression were conducted to explore heterogeneity and were interpreted as exploratory analyses. The subgroup variables (intervention characteristics within exercise‐based interventions, publication period, and geographic region) were prespecified a priori, as they may reflect differences in intervention characteristics, reporting standards, and contextual factors that could influence treatment effects. Subgroup analyses were restricted to exercise‐based intervention studies included in the meta‐analysis. Subgroup analyses were performed based on three predefined characteristics:

i. Intervention characteristics: Studies were categorized within exercise‐based interventions according to protocol characteristics (when applicable).
ii. Publication period: Studies were stratified into three time periods (2014–2016, 2017–2019, and 2020–2024) to assess temporal trends in treatment effects.
iii. Geographic region: Studies were grouped by the geographic region (Asia, Europe, Americas, and Africa) to evaluate the potential regional variations in treatment effectiveness.
iv. Effect size evolution by year and region: For each subgroup analysis, we calculated pooled effect estimates and compared between‐group differences using the Q‐test for subgroup heterogeneity.

### 2.14. Meta‐Regression Analysis

Meta‐regression was conducted as an exploratory analysis to investigate whether treatment duration (in weeks) explained part of the between‐study heterogeneity among exercise‐based intervention studies.

A weighted least squares regression model was employed, in which the SMD (Hedges’ g) was specified as the dependent variable and treatment duration as a continuous predictor. Each study was weighted according to the inverse variance of its effect size, thereby reflecting its precision. The model incorporated effect size as the outcome variable, treatment duration (in weeks) as the independent variable, inverse‐variance weights, and a constant term (intercept).

The strength of the association was evaluated using the regression coefficient (β) and its 95% confidence interval, the proportion of variance explained (*R* ^2^), and the statistical significance of the slope (*p* value). Given the limited number of included studies, the results of the meta‐regression were interpreted with caution and regarded as exploratory and hypothesis‐generating rather than confirmatory.

Heterogeneity was quantified using the Q statistic and the *I* ^2^ index. All statistical analyses were conducted in Python (Version 3.11), using the *statsmodels* and *pandas* libraries for meta‐regression and *matplotlib* and *seaborn* for data visualization. Terminology and abbreviations were standardized throughout the manuscript to enhance clarity and consistency.

### 2.15. Sensitivity Analysis

Sensitivity analyses were conducted to assess the robustness of the findings. Given the heterogeneity and limited number of studies, sensitivity was primarily evaluated through comparison of effect estimates across subgroups and inspection of the consistency of results. These analyses were interpreted cautiously and considered exploratory.

## 3. Results

### 3.1. Study Identification and Selection Process

During the process of identifying and selecting articles, a total of 2847 articles were found in various electronic databases (MEDLINE/PubMed: 305; LILACS and IBECS: 27; EBSCO: 1412; Web of Science: 404; Scopus: 699). After removing duplicates, 1630 articles remained, and their titles were reviewed. A title and abstract screening of 1630 articles was conducted to determine whether the selected studies met the inclusion criteria. In the end, 33 articles met these criteria and were assessed in full text. After reviewing the full text, 31 studies were ultimately included in this literature review.

**FIGURE 1:**
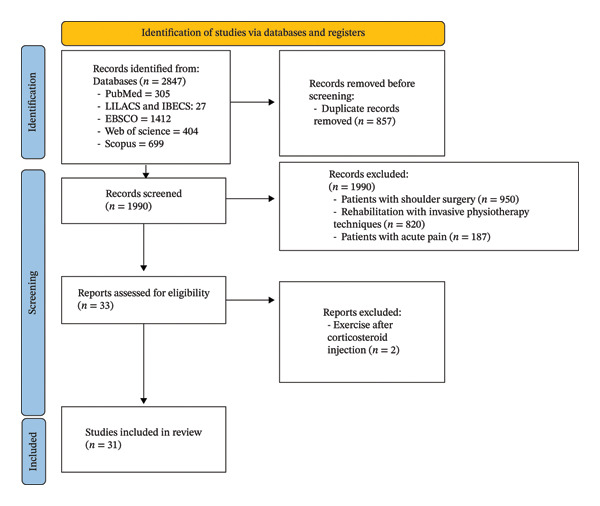
Flow diagram illustrating the study selection process.

The study selection process is illustrated in the flow diagram in Figure 1.

### 3.2. General Characteristics of the Included Studies

This systematic review included 31 studies [48–78]. The publication period ranged from 2010 to 2024, with the highest number of studies published in 2024.

The total sample size across the 31 included studies amounted to 3499 patients. The study by Chan et al. [63] had the largest population (*n* = 24,417), while the study by Jones et al. [49] had the smallest number of participants (*n* = 12).

All studies included both male and female participants. The age range across the studies was broad, spanning from 18 years and older; however, in some studies, this item was not specified [63].

In fact, in the study by Ouyang et al. [75], Chen et al. [77], and Yang et al. [73], a Mendelian randomization approach was used, relying on publicly available GWAS data, and no primary data from individual participants were collected.

The general characteristics of studies included are showed in Table 2, while study design, focus area, intervention/variables investigated, and main outcome measures are presented in Supporting Information B.

**TABLE 2:** General characteristics of the included studies and sample.

Author	Year	Country	Study design	Intervention	Sample	Average age (years ± SD)	Gender
Salek et al. [50]	2010	Bangladesh	Observational, case–control	Association between triglyceride and blood glucose levels and FS	*n* = 60	56.77 ± 8.07	Male Female

Jones et al. [49]	2013	United Kingdom	Qualitative study design using semistructured interviews.	Elucidate the experiences and perceptions of individuals living with primary FS and their treatment priorities	*n* = 12	55 ± 7.5	Male 41.7% Female 58.3%

Sung et al. [52]	2014	South Korea	Case–control study	Identify the association between serum lipid levels and FS	*n* = 12,000	53.93 ± 8.63	Male Female

Ding et al. [53]	2014	China	Cross‐sectional observational study	Prevalence of anxiety and depression risk in patients with FS	*n* = 124	52.01 ± 6.53	Male 37.1% Female 62.9%

Russell et al. [54]	2014	United Kingdom	Randomized controlled trial	Home exercise program vs. scapulothoracic–glenohumeral complex exercise program vs. mobilizations + stretching	*n* = 75	55 ± 7.5	Male Female

Gutierrez Espinosa et al. [55]	2015	Chile	Randomized controlled trial	CPT and exercise	*n* = 57	54 ± 2	Male Female

Ali et al. [56]	2015	Pakistan	Randomized controlled clinical trial	General exercises + manual therapy (Maitland mobilization)	*n* = 43	42.5 ± 8.75	Male Female

Celik et al. [57]	2015	Turkey	Randomized controlled clinical trial	Joint mobilization, stretching, and home exercises for scapular retraction and rotator cuff strengthening vs. stretching	*n* = 30	54.5 ± 0.15	Male 25% Female 75%

Bagheri et al. [58]	2016	Iran	Cross‐sectional study	Evaluate pain, disability, quality of life, and associated factors in patients with FS.	*n* = 120	52 ± 16.25	Male 30.8% Female 69.2%

Schiefer et al. [59]	2016	Brazil	Case control	Prevalence of hypothyroidism in patients with FS	*n* = 244	54.28 ± 13.64	Male 71.3% Female 28.7%

Horst et al. [60]	2017	Poland	Double‐blind randomized controlled trial	Strengthening exercises vs. proprioceptive neuromuscular facilitation (PNF)	*n* = 66	45.5 ± 16.5	Male 62.1% Female 37.9%

Robinson et al. [61]	2017	United Kingdom	Randomized controlled trial	Physiotherapy and active‐assisted and isometric home exercises vs. active‐assisted and isometric exercises alone	*n* = 41	56.55 ± 1.95	Male 31.7% Female 68.3%

Safran et al. [62]	2017	Israel	Prospective cohort study	Prevalence of glucose intolerance in patients with FS	*n* = 50	47.5 ± 6.25	Male 46% Female 54%

Chan et al. [63]	2017	United States	Case series; prognosis study	Correlation between cumulative HbA1c levels in diabetes and FS	*n* = 24,417	*x*	Male 48.3% Female 51.7%

Elhamed et al. [64]	2018	Egypt	Randomized controlled clinical trial	Traditional treatment (including home program) + strengthening exercises lower fibers trapezius	*n* = 30	25.06 ± 3.36	Male Female

Toprak et al. [65]	2018	Turkey	Cross‐sectional observational study	Examine anxiety, depression, sleep quality, and quality of life in patients with FS	*n* = 148	59.32 ± 13.91	Male 27.6% Female 72.4%

Rai et al. [66]	2019	India	Cross‐sectional study	Prevalence of prediabetes in FS.	*n* = 120	49.65 ± 4.8	Male 65.2% Female 34.8%

Ebrahimzadeh [67]	2019	Iran	Cross‐sectional study	Assess the prevalence and effect of depression and anxiety in FS.	*n* = 135	52 ± 16.25	Male 30.8% Female 69.2%

Mohamed et al. [68]	2020	Egypt	Randomized controlled trial	Dynamic scapular recognition exercises with a wireless biofeedback system.	*n* = 60	51.0 ± 6.02	Male 43.3% Female 56.7%

Park et al. [69]	2020	Korea	Case–control study	Association between fasting glucose levels and FS.	*n* = 604	52.1 ± 7.2	Male Female

Lin et al. [70]	2022	China	Randomized controlled clinical trial	Passive mobilizations + PNF	*n* = 48	50 ± 5	Male 45.8% Female 54.2%

Jesic et al. [71]	2022	Slovenia	Prospective study	Prevalence of elevated blood glucose in FS.	*n* = 26	49.8 ± 11.0	Male Female

Razzaq et al. [51]	2022	Pakistan	Comparative study	Muscle energy technique vs. Mulligan mobilization with movement.	*N* = 64	49.55 ± 7.89	Male Female

Wang et al. [72]	2023	China	Randomized controlled clinical trial	Neuromuscular exercises vs. strengthening exercises (isometric and isotonic with Theraband and dumbbells).	*n* = 40	54.23 ± 5.55	Male 52.5% Female 47.5%

Yang [73]	2023	China	Mendelian randomization study	Causal relationship between socioeconomic level, obesity, individual behaviors, diabetes, and the risk of FS.	*x*	*x*	Male Female

Romeo et al. [74]	2023	United States	Retrospective review conducted on a prospectively enrolled database	Analyze the influence of anxiety, hypothyroidism, and hyperlipidemia on FS.	*n* = 56	54.7 ± 7.7	Male 28.6% Female 71.4%

Ouyang et al. [75]	2024	China	Bidirectional Mendelian randomization study	Relationship between anxiety disorders and FS.	*x*	X	X

Sheikh et al. [76]	2024	Pakistan	Randomized clinical trial	Moderate physical activity vs. conventional physiotherapy with passive shoulder mobilizations and active stretching.	*n* = 44	52.16 ± 5.31	Male 13.6% Female 86.4%

Chen et al. [77]	2024	China	Mendelian randomization study	Analysis of the human plasma proteome (Mendelian randomization study)	*x*	X	X

Hamed et al. [78]	2024	Spain	Cross‐sectional observational study	Relationship between metabolic biomarkers (liver enzymes and thyroid function) and shoulder pain and functionality in patients with FS.	*n* = 32	55.23 ± 9.56	Male 31.2% Female 68.8%

Mertens et al. [48]	2024	Belgium	Cross‐sectional study	Compare blood glucose levels between patients with FS and asymptomatic individuals.	*n* = 70	53.00 ± 8.00	Male 37.1% Female 62.9%

*Note:* CPT = Conventional physiotherapy; *N* = number; *x* = missing data.

Abbreviations: FS = Frozen shoulder, PNF = proprioceptive neuromuscular facilitation, RCT = randomized controlled clinical trial, SD = standard deviation.

### 3.3. Risk of Bias in the Included Studies

The risk of bias of the included studies varied according to study design and is summarized in Tables 3, 4, 5.

**TABLE 3:** Risk of bias assessment of randomized controlled trials using the Cochrane Risk of Bias tool (RoB 1).

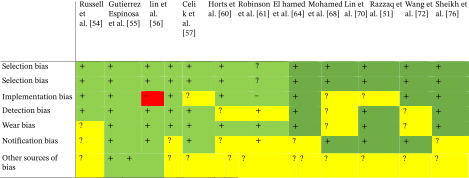

*Note:* The table details the risk of bias in the included articles. The different colors indicate the level of risk of bias for each study: high risk (red), unclear risk (yellow), and low risk of bias (green). Overall, the risk of bias was lower in exercise/rehabilitation trials and more frequently high/unclear in metabolic and psychological studies, largely reflecting limitations inherent to noninterventional designs. This pattern was considered when interpreting domain‐specific findings and informed a cautious framing of conclusions for metabolic and psychological evidence.

**TABLE 4:** Risk of bias assessment of observational studies using a domain‐based adaptation of the Newcastle–Ottawa scale (NOS).

Study	Design	Selection bias	Comparability	Outcome/exposure	Overall risk of bias
Ding et al. [53]	Cohort	Low	Low	Low	**Low**
Toprak et al. [65]	Cohort	Low	Low	Low	**Low**
Romeo et al. [74]	Cohort	Low	Low	Low	**Low**
Schiefer et al. [59]	Case–control	Low	Low	Low	**Low**
Park et al. [69]	Case–control	Low	Low	Low	**Low**
Bagheri et al. [58]	Case–control	Low	Moderate	Low	**Moderate**
Salek et al. [50]	Case–control	Low	Moderate	Low	**Moderate**
Sung et al. [52]	Case–control	Low	Moderate	Low	**Moderate**
Chan et al. [63]	Retrospective cohort	Low	Moderate	Low	**Moderate**
Mertens et al. [48]	Cross‐sectional	Moderate	Moderate	Low	**Moderate**
Hamed et al. [78]	Cross‐sectional	High	Moderate	Low	**Moderate–High**
Safran et al. [62]	Case series	High	High	Moderate	**High**
Rai et al. [66]	Case series	High	High	Moderate	**High**
Ješić et al. [71]	Prospective study	High	High	Moderate	**High**

*Note:* Risk of bias was evaluated across three domains: selection (representativeness of the sample and selection of participants), comparability (control for confounding variables), and outcome/exposure (assessment and measurement of outcomes or exposures). Each domain was classified as low, moderate, or high risk of bias. An overall risk of bias judgment was assigned based on the combined domain ratings. Low: low risk of bias. Moderate: moderate risk of bias. Moderate–High: moderate to high risk of bias. High: high risk of bias.

**TABLE 5:** Risk of bias assessment of Mendelian randomization studies based on instrumental variable assumptions.

Study	Relevance (IV strength)	Independence (no confounding)	Exclusion restriction (no pleiotropy)	Sensitivity analyses	Overall judgment
Ouyang et al. [75]	Met (*F* > 10, SNP selection)	Likely met (confounders addressed)	Met (MR‐Egger, MR‐PRESSO, no pleiotropy)	Comprehensive (leave‐one‐out, multiple methods)	**Low risk**
Chen et al. [77]	Met (genome‐wide significance, *F* > 10)	Likely met (*cis*‐pQTLs, validation datasets)	Partially met (pleiotropy detected in T1D)	Extensive (reverse MR, colocalization, validation)	**Moderate risk**
Yang et al. [73]	Met (*F* > 10, strong SNPs)	Likely met (GWAS datasets, standard MR design)	Met (no pleiotropy detected)	Adequate (MR‐Egger, MR‐PRESSO, leave‐one‐out)	**Moderate risk**

*Note:* Mendelian randomization studies were assessed qualitatively based on the core instrumental variable assumptions: relevance (strength of genetic instruments), independence (absence of association with confounders), and exclusion restriction (absence of horizontal pleiotropy). Sensitivity analyses, including MR‐Egger, MR‐PRESSO, leave‐one‐out, and additional validation approaches, were considered to evaluate the robustness of the findings. Studies were classified as low, moderate, or high risk of bias according to the extent to which these assumptions were satisfied. The categories correspond to the risk of bias assessment of the Mendelian randomization studies: Low Risk: low risk of bias. Moderate Risk: moderate risk of bias.

For RCTs (Table 3), the overall risk of bias was generally low to moderate. Most studies assessing exercise‐based interventions demonstrated a low risk of bias across key domains, including randomization, outcome measurement, and reporting. In particular, studies by Russel et al. [54], Gutierrez et al. [55], Ali et al. [56], Celik et al. [57], Horst et al. [60], Mohamed et al. [68], Romeo et al. [74], and Sheikh et al. [76] showed the lowest risk of bias. However, some studies presented unclear risk in at least one domain, mainly related to allocation concealment or blinding procedures.

For observational studies (Table 4), risk of bias was assessed using a domain‐based adaptation of the NOS, including selection, comparability, and outcome/exposure domains. Cohort and case–control studies generally demonstrated low risk of bias, reflecting appropriate participant selection, control for confounding variables, and reliable outcome assessment. Cross‐sectional studies were typically rated as having moderate risk of bias, mainly due to limitations in comparability and potential selection bias. Case series and small uncontrolled studies showed a higher risk of bias, particularly due to the absence of comparison groups and lack of adjustment for confounders. Overall, most observational studies were classified as having low to moderate risk of bias, although findings from weaker designs should be interpreted with caution.

For Mendelian randomization studies (Table 5), risk of bias was evaluated qualitatively based on the core instrumental variable assumptions: relevance, independence, and exclusion restriction. Most studies satisfied the relevance assumption, supported by strong genetic instruments. Independence and exclusion restriction assumptions were generally addressed through sensitivity analyses, including MR‐Egger, MR‐PRESSO, and leave‐one‐out analyses. Overall, the included Mendelian randomization studies were considered to have low to moderate risk of bias, although potential pleiotropy and residual confounding cannot be completely excluded.

Across all study designs, studies investigating metabolic factors tended to present a higher risk of bias compared to interventional studies, mainly due to confounding and selection bias inherent to nonrandomized designs. Therefore, these findings were interpreted as associative rather than causal and were considered with caution in the overall synthesis of evidence.

### 3.4. Variables Investigated and Instruments of Measurement

The studies included in this systematic review examined the factors influencing the pain and function, focusing on three main areas: physical exercise, metabolic factors, and lifestyle combined with psychological asset. These trials also assessed the effect of such variables on the body’s biorhythm in patients with FS.

The body’s biorhythm, also known as the circadian rhythm, is a biological cycle of approximately 24 h that regulates physiological functions such as sleep, eating, hormonal activity, and body temperature. Its regulation depends on the suprachiasmatic nucleus (SCN) in the hypothalamus, which responds to external stimuli, mainly light. Disruptions in these rhythms can affect the immune system and increase the risk of various diseases [79, 80]. For example, shift workers, who frequently experience circadian misalignment, have a higher incidence of metabolic disorders, cardiovascular disease, and certain cancers (e.g., breast and colorectal cancer) [4]. Additionally, sleep deprivation and irregular sleep–wake cycles have been linked to increased inflammatory markers such as C‐reactive protein (CRP) and interleukin‐6 (IL‐6), which can impair immune function and exacerbate chronic pain conditions [79].

Table 6 detailed the characteristics of the groups, the length of intervention, variables of interest, and outcome measurements.

**TABLE 6:** Groups, length of intervention, variables, and outcome measurement specification.

Author	Year	Sample	Groups		Length of intervention (weeks)	Variables	Instrument of measurement
			Experimental group	Control group			
Salek et al. [50]	2010	*n* = 60	Not reported	Not reported	Not reported	Triglyceride levels, fasting blood glucose, and 2 h postprandial glucose, HbA1c	Laboratory test

Jones et al. [49]	2013	*n* = 12	Not reported	Not reported	Not reported	Pain Function Confusion/anxiety	No standardized scales were used; data were collected through interviews.

Sung et al. [52]	2014	*n* = 1200	*G*1 = FS *n* = 300	*G*2 = Controls *n* = 900	Not reported	Total cholesterol, LDL, HDL, triglycerides, non‐HDL cholesterol levels.	Laboratory test

Ding et al. [53]	2014	*n* = 154	GE = FS *n* = 124	GC = healthy *n* = 130	Not reported	Pain, shoulder function, disability.	VAS SST, SPADI HAQ

Russell et al. [54]	2014	*n* = 75	*G*1 = Exercises (circuit training [12 stations, ROM + scapular + trunk exercises]), *n* = 25 *G*2 = Individual physiotherapy (mobilization, soft tissue, stretching) *n* = 24	*G*3 = Home‐based exercises, *n* = 26	6 weeks (2×/week; ∼50 min/session)	Pain Functionality	Constant–Murley score, Oxford Shoulder Score

Gutierrez Espinosa et al. [55]	2015	*n* = 57	*G*1 = Exercise + mobilization (posterior glide mobilization)	*G*2 = Physiotherapy + active exercises (cycle ergometer warm‐up + posterior glide mobilization)	5 weeks	Pain and functionality	VAS

Ali et al. [56]	2015	*n* = 44	*G*1 = Mobilization exercise (Maitland II–III mobilizations (30 s × 5 sets); stretching + pendulum + ROM)	*G*2 = Exercise (home exercises daily)	5 weeks (3×/week; ∼45 min/session)	Pain Functionality	VAS

Celik et al. [57]	2015	*n* = 30	*G*1 = Joint mobilization + stretching (mobilization [Grades I–IV; 2–3 oscillations/sec, 1–2 min × 3–4 reps]; cyclic stretching [20 s hold, 10 reps/direction]; home program [2×/day]), *n* = 15	*G*2 = Stretching only, both with a home exercise program, *n* = 15	6 weeks (3×/week; ∼50 min/session)	Pain Functionality	VAS DASH

Bagheri et al. [58]	2016	*n* = 120	*G*1 = n120	Not reported	Not reported	Pain, disability, and quality of life	VAS DASH HAD.

Schiefer et al. [59]	2016	*n* = 244	*G*1 = FS *n* = 93	*G*2 = controls *n* = 151	Not reported	TSH and free T4 levels, elevation range, external and internal rotation, symptom duration, presence of hypothyroidism, diabetes mellitus, systemic arterial hypertension, cardiovascular disease.	Goniometer, immunometric assay

Horst et al. [60]	2017	*n* = 66	*G*1 = Group activity (task‐oriented ADL training vs manual therapy + PNF; resistance, aerobic, cryotherapy) *n* = 33	*G*2 = conventional therapy (resistance, aerobic, cryotherapy) *n* = 33	2 weeks (daily; 30 min/session)	Pain Functionality	McGill Pain Questionnaire

Robinson et al. [61]	2017	*n* = 41	*G*1 = Supervised physiotherapy (mobilization + stretching + advice) = 20	*G*2 = Home exercise program: (wall slides, stick exercises, stretches, scapular setting) = 21	4 weeks (1x/week + daily home)	Pain Functionality	VAS OSS

Safran et al. [62]	2017	*n* = 50	*G*1 = FS *n* = 50	Not reported	Not reported	Fasting blood glucose levels	OGTT

Chan et al. [63]	2017	*n* = 24,417	Not reported	Not reported	Not reported	Cumulative HbA1c levels, age, body mass index, diagnosis of FS	Laboratory test

Elhamed et al. [64]	2018	*n* = 30	*G*1 = Conventional physiotherapy	*G*2 = Physiotherapy + exercise (Prone cobra and V‐raise (10 reps, 3–4s hold); + mobilization + ultrasound + ROM exercises)	4 weeks (3×/week)	Pain, function	VAS A‐T TEST

Toprak et al. [65]	2018	*n* = 148	GE = FS *n* = 76	GC = Healthy control group = 72	Not reported	Pain, anxiety, depression, sleep quality, and quality of life (QoL) (poor sleep, ↑ anxiety, ↓ QoL.	VAS, BAI, BDI, PSQI, WHOQoL‐BREF

Ebrahimzadeh et al. [67]	2019	*n* = 120	*G*1 = FS *n* = 120	Not reported	Not reported	Range of motion, pain, and disability (↑ pain and disability; high prevalence)	VAS DASH HADS

Rai et al. [66]	2019	*n* = 135	*G*1 = FS *n* = 135	Not reported	Not reported	Pain Blood glucose levels	SPADI

Mohamed et al. [68]	2020	*n* = 60	GE = scapular exercise (wireless biofeedback training targeting scapular upward rotation) *n* = 30	GC = exercise active *n* = 30	8 weeks	Pain Functionality	SPADI

Park et al. [69]	2020	*n* = 604	*G*1 = FS *n* = 151	*G*2 = controls *n* = 453	Not reported	Fasting blood glucose levels, body mass index, serum lipid profiles, thyroid hormone levels, high‐sensitivity C‐reactive protein.	Laboratory test

Lin et al. [70]	2022	*n* = 48	PNF group (diagonal patterns, hold‐relax, rhythmic stabilization) *n* = 24	Manual therapy group ([Maitland I–IV]; ESWT + ultrasound) *n* = 24	4 weeks (daily adjunct therapies)	Pain Functionality	VAS

Jesic et al. [71]	2022	*n* = 26	*G*1 = FS *n* = 26	Not reported	104 weeks	Time from symptom onset to treatment initiation, comorbid medical diagnoses, blood sugar levels, passive range of motion, functional status	DASH SPADI

Razzaq et al. [51]	2022	*N* = 64	*G*1 = MWM (5 reps × 2 sets) + conventional treatment (pulley, wall climbing, etc.) *n* = 32	*G*2 = MET (5 reps × 2 sets) + conventional treatment (pulley, wall climbing, etc.), *n* = 32	3 weeks (3×/week; sets/reps defined)	Pain Functionality	NPRS SPADI

Wang et al. [72]	2023	*n* = 40	GE = NMES + regular physiotherapy (6 exercises, 10‐s work/10‐s rest × 8 reps; 5 levels based on performance); plus mobilization, stretching, AROM), *n* = 20	GC = Strengthening exercises + regular physiotherapy *n* = 20	8 weeks (5×/week; ∼65 min/session)	Pain Functionality	VAS

Yang et al. [73]	2023	No applicable	Not reported	Not reported	Not reported	Type 1 diabetes Type 2 diabetes Smoking Alcohol Nutritional intake	Laboratory test

Romeo et al. [74]	2023	*n* = 56	*G*1 = FS	Not reported	Minimum 52 weeks	Pain Functionality (worse improvement in PROMs)	Extremity Computer Adaptive Test Version 2.0 (P‐UE), VAS

Ouyang et al. [75]	2024	Not applicable (two‐sample Mendelian randomization study)	Not reported	Not reported	Not reported	Causal association between anxiety and FS (anxiety ↑ risk of FS)	Not applicable (genetic study); uses data from existing datasets for genetic analysis.

Sheikh et al. [76]	2024	*n* = 44	*G*1 = CPT (treadmill walking (30 min, 3–6 METs, 50%–70% HRmax); CPT (TENS, mobilization, stretching) *n* = 22	*G*2 = CPT *n* = 22	6 weeks (5×/week; 60 min/session)	Pain Functionality	NPRS DASH

Chen et al. [77]	2024	Not reported	Not reported	Not reported	Not reported	Fasting glucose, Type 1 diabetes, Type 2 diabetes, fasting insulin, glycated hemoglobin, 2‐h postprandial glucose, plasma proteins. IVW, weighted median, MR‐Egger methods, GWAS	IVW, weighted median, MR‐Egger methods, GWAS

Hamed et al. [78]	2024	*n* = 32	*G*1 = FS *n* = 32	Not reported	Not reported	AST, ALT, GGT, TSH, pain, shoulder functionality. SPADI, ASES scores	SPADI, ASES scores

Mertens et al. [48]	2024	*n* = 70	*G*1 = FS *n* = 35	*G*2 = control *n* = 35	Not reported	Muscle strength (abduction, external and internal rotation), scapular kinematics, proprioception, blood glucose levels (HbA1c), pain, and disability.	Handheld dynamometer, inclinometer, SPADI, and McGill Pain questionnaires

*Note:* Number (n), Group 1(G1), Group 2 (G2), Disabilities of the Arm, Shoulder and Hand Questionnaire (DASH), World Health; Organization Quality of Life Scale short form (WHOQoL‐BREF), Shoulder Score (OSS); hemoglobin A1c – (hBa1C), aspartate aminotransferase, enzyme (AST), alanine aminotransferase, enzyme (ALT), gamma‐glutamyl transferase, enzyme (GGT), conventional physiotherapy (CPT), association test, genetic (A‐T TEST), inverse‐variance weighted analysis (IVW).

Abbreviations: ASES, American Shoulder and Elbow Surgeons; CG, control group; EG, experimental group; GWAS, genome‐wide association study; HADS, Hospital Anxiety and Depression Scale; HAQ, Health Assessment Questionnaire; HDL, high‐density lipoprotein; LDL, low‐density lipoprotein; MET, muscle energy technique; MWM, mobilization with movement; NMES, neuromuscular electrical stimulation; NPRS, numeric pain rating scale; OGTT, oral glucose tolerance test; PNF, proprioceptive neuromuscular facilitation; PSQI, Pittsburgh Sleep Quality Index; SPADI, Shoulder Pain and Disability Index; SST, simple shoulder test; TSH, thyroid‐stimulating hormone; VAS, visual analog scale.

### 3.5. Main Findings

The characteristics of the main findings of the included studies are presented in Tables 7, 8, and 9, respectively, for physical exercise, metabolic, and psychological factors.

**TABLE 7:** Main findings of the included studies on physical exercise (*n* = 12).

Author	Main findings	Statistical significance/notes
Russell et al. [54]	Group exercise classes are strongly supported as part of FS treatment.	Strong evidence for physiotherapy, especially in group settings.
Gutierrez et al. [55]	Group A (mobilizations): +46.3° in external rotation; Group B (physio + exercise): +18.1° in external rotation.	*p* < 0.0001 (ROM difference); pain and function also improved significantly in Group A.
Ali et al. [56]	Both groups improved in pain and function; no significant difference between groups.	*p* < 0.001 within groups; *p* > 0.05 between groups; VAS ↓2.23 (Group 1), ↓2.33 (Group 2); SPADI ↑22 vs. ↑23.
Celik et al. [57]	Mobilization + stretching more effective than stretching alone for ROM and function.	External rotation and abduction improved more in the combined group.
Horst et al. [60]	Activity‐focused therapy led to better ADL performance and functional outcomes vs. conventional.	Significant improvements after 10 days and at 3‐month follow‐up.
Robinson et al. [61]	Both groups improved (OSS, ROM), but no differences between groups.	Improvements sustained at 4 weeks and 1 year.
Elhamed et al. [64]	Strengthening lower trapezius improved scapular tilt in multiple positions.	MANOVA *p* < 0.05; only Group B (with exercise) showed significant pre‐/postdifferences.
Mohamed et al. [68]	Dynamic scapular recognition exercises improved shoulder movement and function.	Benefits maintained for up to 6 months.
Lin et al. [70]	PNF more effective than conventional mobilization for pain and ROM.	Significant reductions in pain and improvements in ROM.
Razzaq et al. [51]	Both MET and MWM were effective; MWM superior for pain, ROM, function.	MWM showed greater clinical improvements overall.
Wang et al. [72]	Neuromuscular exercise group had better pain and ROM outcomes than strengthening group.	Time had a significant effect on improvements.
Sheikh et al. [76]	Moderate physical activity + therapy led to superior outcomes vs. therapy alone.	Significant improvement in pain, ROM, and functionality in Group A.

Abbreviations: ADL, Activity of daily living; FS, frozen shoulder; MET, muscle energy Technique; MWM, mobilization with movement; OSS, Oxford shoulder score; PNF, proprioceptive neuromuscular facilitation; ROM, range of motion; SPADI, Shoulder Pain and Disability Index; VAS, visual analog scale.

**TABLE 8:** Results of the included studies on metabolic factors (*n* = 12).

Author	Main findings	Statistical significance/notes
Salek et al. [50]	FS group had higher fasting glucose, 2‐h postbreakfast glucose, HbA1c, and triglycerides.	Fasting glucose (*p* = 0.012), 2‐h glucose (*p* < 0.01), HbA1c (*p* < 0.05), TG (*p* < 0.001).
Sung et al. [52]	Significant association between FS and lipid profile (TC, LDL, HDL, non‐HDL).	TC, LDL, non‐HDL (*p* < 0.001); HDL (*p* = 0.001).
Schiefer et al. [59]	Higher prevalence of hypothyroidism in FS patients; higher TSH linked to severe/bilateral FS.	Hypothyroidism (*p* = 0.001); TSH (*p* = 0.05).
Safran et al. [62]	8% of FS patients had prediabetes; mean fasting glucose = 4.5 mmol/L; 2 h glucose = 5.5 mmol/L.	No *p*‐values reported; small sample.
Chan et al. [63]	No significant differences in the BMI or hypothyroidism between groups.	BMI (*p* = 0.29); hypothyroidism not significant.
Rai et al. [66]	15.5% had prediabetes, 27.4% diabetes; FS patients advised to undergo glucose testing.	Clinical recommendation; no *p*‐values provided.
Park et al. [69]	FS associated with fasting glucose, hypercholesterolemia, and hs‐CRP.	Fasting glucose (*p* < 0.001); overall association (*p* < 0.030).
Jesic et al. [71]	Most FS patients had normal glucose; recommended glucose screening despite no ROM link.	No significant correlation between glucose and sick leave duration.
Yang et al. [73]	Type 1 diabetes increases the risk of FS. No significant causal effect found for Type 2 diabetes, BMI, or waist circumference.	Mendelian randomization study
Chen et al. [77]	Mendelian randomization showed causal link between fasting glucose, T1D, T2D, and FS.	Positive causal relationship via IVW analysis.
Hamed et al. [78]	Metabolic dysfunction (insulin resistance, lipid disorders, inflammation) linked to FS severity.	Functional limitation and pain associated with metabolic profile.
Mertens [48]	No clinically relevant differences in HbA1c between FS patients and asymptomatic controls.	HbA1c: 5.28% vs. 4.86%; *p* = 0.0056; Hedges’ *g* = 0.78 (moderate).

*Note:* Hemoglobin A1c (HbA1c), diabetes Type 1 (T1D), diabetes, Type 2 (T2D).

Abbreviations: BMI, Body mass index; HDL, high‐density lipoproteins; hs‐CRP, high‐sensitivity C‐reactive protein; IVW, inverse‐variance weighted analysis; LDL, low‐density lipoproteins; non‐HDL, non–high‐density lipoproteins; ROM, range of motion; TC, total cholesterol; TSH, thyroid‐stimulating hormone.

**TABLE 9:** Results of the included studies on psychological factors (*n* = 7).

Author	Main findings	Statistical significance/notes
Jones et al. [49]	Patients with FS‐reported pain, disability, anxiety, diagnostic delays, and lack of clear information.	Specialist referral helped reduce anxiety and improved perceived outcomes.
Ding et al. [53]	Depression (28.2%) and anxiety (24.2%) prevalent in FS; both linked to greater pain, disability, and sleep issues.	HADS scores significantly higher in patients with psychological disorders.
Bagheri et al. [58]	VAS pain correlated with depression; DASH with anxiety, ROM, and sex. PCS/MCS affected by age and depression scores.	DASH independently impacted by HADS‐A and ROM; multivariable analysis supports findings.
Toprak et al. [65]	No significant difference in the BDI between FS and controls. Mean VAS: 6.03, BAI: 18.4, PSQI: 15 in the FS group.	Suggests subclinical mood disturbances despite the lack of depression diagnosis.
Ebrahimzadeh et al. [67]	Depression in 77%, anxiety in 27% of FS patients. Depression associated with limited internal/external rotation and worse DASH/VAS.	DASH correlated with anxiety/depression severity in multivariable analysis.
Romeo et al. [74]	Anxiety associated with worse pROM improvements. Female sex and manual labor linked to better outcomes.	Anxiety (PROMIS‐UE b: 4.13, *p* = 0.03); hypothyroidism and ethnicity also affected PROMs.
Ouyang et al. [75]	Anxiety increased the likelihood of developing FS.	Statistical model confirmed association (exact *p* value not reported).

*Note:* Disability od the Arm, Hand, and Shoulder score (DASH), Physical Component Summary/Mental Component Summary, from SF‐36 Health Survey Short Form (PCS/MCS).

Abbreviations: BAI, Back Anxiety Index; BDI, Back Depression Index; HADS, Hamilton Anxiety and Depression Score; pROM, passive range of motion; PROMIS‐UE, Patient‐Reported Outcomes Measurement Information System‐Upper Extremity ain, disability, and sleep disturbances [89]. Importantly, psychological factors particularly anxiety and depression were consist; PROMs, patient‐reported outcome measures; PSQI, Pittsburgh Sleep Quality Index; ROM, range of motion; VAS, visual analog score.

#### 3.5.1. Physical Exercise

##### 3.5.1.1. Meta‐Analysis of Exercise‐Based Interventions

**FIGURE 2:**
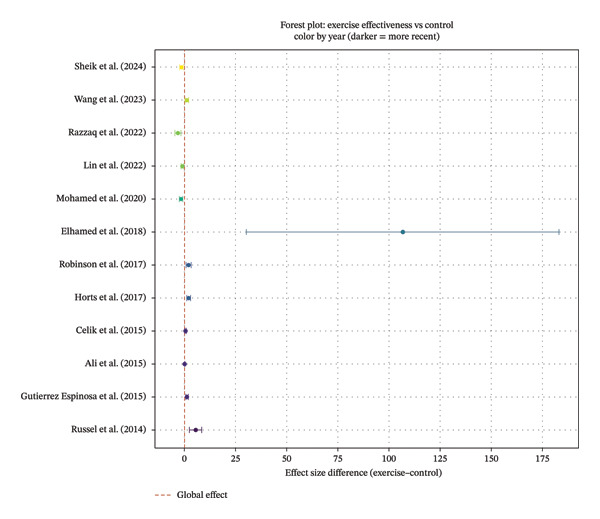
Diagram of forest plot. Overall layout: The horizontal axis represents the difference in effect size (exercise minus control). The vertical axis lists each study, labeled with the author and year. Data points and error bars: Each study is depicted as a point with horizontal error bars showing the 95% confidence interval for the effect difference. The effect size difference is calculated as the change in the exercise group minus the change in the control group. Color coding: Data points are color‐coded according to the year of publication. Studies are ordered chromatically from most recent to oldest, following a transition from yellow and greenish colors (most current studies) to blue shades (oldest studies). This allows us to visualize possible time trends in effect sizes. Global effect summary: A red dashed vertical line is drawn at the global weighted effect size (here, an effect size of 0.10), summarizing the overall effect when considering all the studies. Heterogeneity information. The heterogeneity statistics (Q, *I* ^2^, and *p* value) indicate that a high level of variation exists among the studies (with *I* ^2^ around 91.50% and a significant *p* value), suggesting that the effects differ substantially across studies. Therefore, the pooled estimate should be interpreted cautiously as an average effect across heterogeneous protocols and study contexts, and heterogeneity exploration (subgroup analysis and meta‐regression) was performed to identify potential drivers of variability. Interpretation: An effect size difference greater than zero (to the right of the zero line) indicates that the exercise intervention is more effective than the control treatment. The global weighted effect of 0.10, with a 95% CI of [0.05, 0.15], suggests a statistically significant but small overall benefit of exercise. The spread of the individual study estimates and their confidence intervals, along with the high heterogeneity, highlight the variability in effects among the different studies; thus, a subgroup analysis were performed.

A quantitative meta‐analysis was conducted including the 12 studies evaluating exercise‐based interventions. Only exercise‐based interventions met the criteria for quantitative synthesis and meta‐analysis; therefore, a forest plot is presented exclusively for this domain (Figure 2), while the remaining evidence streams were synthesized narratively due to substantial methodological heterogeneity. All other evidence streams were synthesized narratively and were not included in the quantitative pooling.

The pooled analysis demonstrated a small but statistically significant effect in favor of exercise interventions (SMD = 0.10; 95% CI [0.05, 0.15]). An effect size greater than zero indicates a benefit of exercise compared to control interventions.

To facilitate clinical interpretation, this effect size (SMD = 0.10) can be considered small according to conventional thresholds. This magnitude suggests a modest improvement in patient‐reported outcomes such as pain and function, indicating that while exercise provides a statistically significant benefit, its clinical impact may be limited when considered in isolation and should be interpreted within a multimodal treatment context.

##### 3.5.1.2. Heterogeneity

Substantial heterogeneity was observed across studies (*I* ^2^ = 91.5%, *p* < 0.001), indicating considerable variability in effect sizes. Therefore, the pooled estimate should be interpreted as an average effect across heterogeneous interventions rather than a single universally applicable effect.

##### 3.5.1.3. Forest Plot Interpretation

Figure 2 presents the forest plot of the included studies. Each study is represented by its SMD and corresponding 95% confidence interval, calculated based on the difference in pre–post‐changes between exercise and control groups.

Most studies showed effects favoring exercise, although the magnitude of benefit varied considerably across studies. The overall pooled estimate supports a modest but consistent benefit of exercise‐based interventions.

##### 3.5.1.4. Overall Interpretation

Overall, exercise‐based interventions appear to provide a small but consistent benefit in improving pain, function, and ROM in patients with FS on factors such as intervention type, intensity, duration, and population characteristics.

These findings support the use of exercise as part of a multimodal treatment approach, while highlighting the need for individualized intervention protocols.

###### 3.5.1.4.1. Subgroup Analysis

**FIGURE 3:**
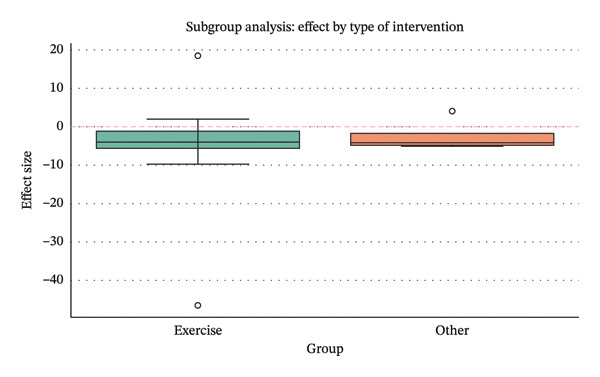
Subgroup analysis of exercise‐based interventions according to intervention characteristics. Effect sizes are expressed as standardized mean differences (SMDs) with 95% confidence intervals. Positive values indicate a benefit in favor of exercise‐based interventions. Description: This figure presents the distribution of effect sizes across different exercise‐based intervention approaches. Given that all included studies involved exercise interventions, comparisons were performed within exercise modalities rather than between exercise and nonexercise interventions. Key points: (I) The *x* axis represents the categories of exercise‐based interventions, while the *y* axis shows the SMD. (II) A horizontal dashed line at zero indicates no effect. (III) The boxplots illustrate the median, interquartile range, and variability within each subgroup. Interpretation: The results show considerable overlap between subgroups, suggesting that no specific exercise modality demonstrates clear superiority over others. The wide variability reflects differences in intervention protocols, supervision, cointerventions, and study populations. Therefore, these findings should be interpreted cautiously, and subgroup analyses considered exploratory.

**FIGURE 4:**
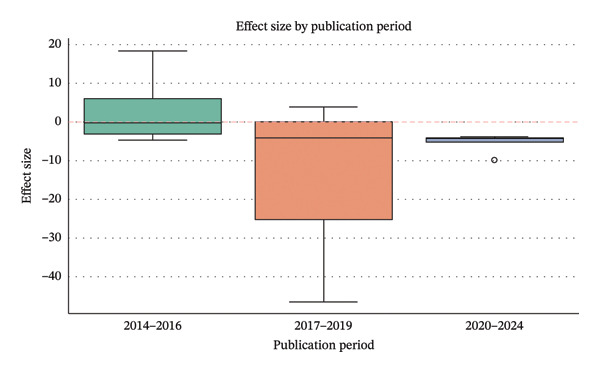
Subgroup analysis of effect sizes by the publication period. Effect sizes are expressed as standardized mean differences (SMDs) with 95% confidence intervals. Positive values indicate a benefit in favor of exercise‐based interventions. Description: This figure presents the distribution of effect sizes across three publication periods (2014–2016, 2017–2019, and 2020–2024). The *x* axis categorizes studies by publication period, while the *y* axis shows the standardized mean difference (SMD). A horizontal dashed line at zero indicates no effect. 2. Key points: (I) The *x* axis categorizes studies by their publication period. (II) The *y* axis displays the effect size. (III) The red dashed line at zero serves as a reference point for determining whether the overall effect is positive or negative. 3. Interpretation: Although some variation in effect sizes across publication periods may be observed, there is substantial overlap between groups, suggesting no clear temporal trend. These findings indicate that the publication period is unlikely to be a major source of heterogeneity. However, this analysis should be interpreted cautiously, as subgroup analyses are exploratory and the number of studies within each category is limited.

**FIGURE 5:**
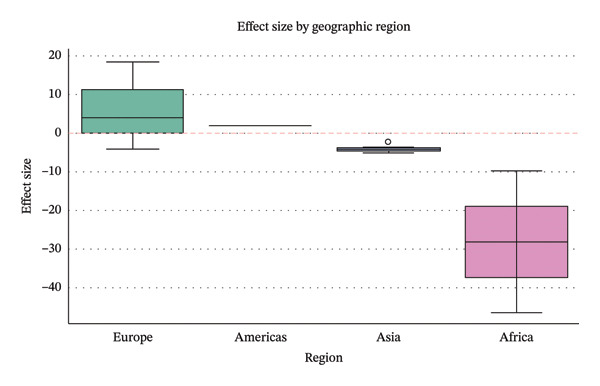
Subgroup analysis of effect sizes by the geographic region. Effect sizes are expressed as standardized mean differences (SMDs) with 95% confidence intervals. Positive values indicate a benefit in favor of exercise‐based interventions. Description: This figure presents the distribution of effect sizes across geographic regions (Africa, Americas, Asia, and Europe). The *x* axis represents the geographic regions, while the *y* axis shows the standardized mean difference (SMD). A horizontal dashed line at zero indicates no effect. 2. Key points: (I) The *x* axis represents broad geographic regions. (II) The *y* axis shows the effect size. (III) Again, the red dashed line at zero indicates no effect. 3. Interpretation: (I) Differences in medians and spreads between regions may suggest that geographical context (and by extension, study context, or population characteristics) could influence the treatment effect. (II) The variability in effect sizes within each region helps explain aspects of the overall heterogeneity across studies.

**FIGURE 6:**
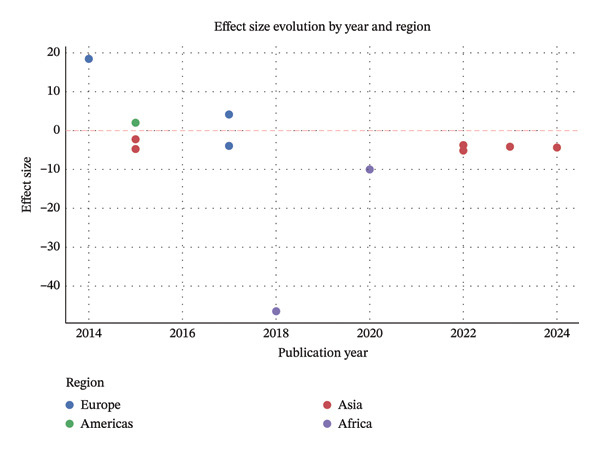
Scatterplot of effect sizes by publication year and geographic region. Effect sizes are expressed as standardized mean differences (SMDs). Positive values indicate a benefit in favor of exercise‐based interventions. Description: This figure presents the distribution of effect sizes across publication years, with each study represented as a point. The *x* axis corresponds to publication year, and the *y* axis represents the standardized mean difference (SMD). Points are color‐coded according to the geographic region. A horizontal dashed line at zero indicates no effect. 2. Key points: (I) The *x* axis represents the publication year, and the *y* axis represents the effect size. (II) The red dashed line at zero provides a reference level for no effect. (III) Differently colored points allow you to see how effect sizes differ by region as well as track any temporal trends. 3. Interpretation: (I) This visualization permits an integrated view of how effect sizes have evolved over time and whether certain regions display consistent trends. (II) It helps identify whether shifts in effect sizes are related to the publication period or if regional differences remain constant over time.

**FIGURE 7:**
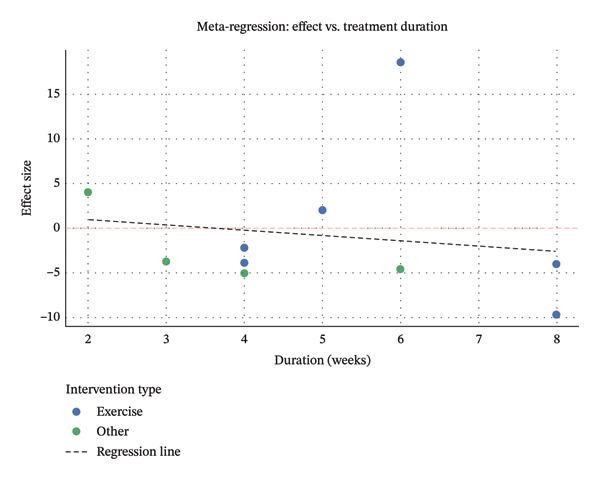
Meta‐regression analysis of treatment duration and effect size. Effect sizes are expressed as standardized mean differences (SMDs). Graph components: *x* axis: Duration (weeks)—shows the treatment duration. *y* axis: effect size—shows the magnitude of the effect. Points: Individual studies colored by intervention type (exercise vs other). Black dashed line: Regression line showing the estimated relationship. Red dashed line: Reference line at zero (no effect). 2. Statistical results: Meta‐regression results: Intercept: 1.98. Slope: −0.58. *p* value (slope): 0.682. *R*‐squared: 0.022 3. Interpretation: (I) The regression line shows a slight negative slope (−0.58), suggesting that for each additional week of treatment, the effect size decreases by 0.58 units. (II) However, this relationship is not statistically significant (*p* = 0.682). (III) The R‐squared value of 0.022 indicates that only 2.2% of the variability in effect sizes is explained by treatment duration. (IV) The intercept of 1.98 represents the estimated effect size when duration is zero (theoretical). (V) The scattered distribution of points and nonsignificant *p* value suggest that treatment duration alone does not explain the heterogeneity in the results. 4. Key findings: (I) There is no strong evidence that treatment duration significantly influences the effect size. (II) The high variability in effects (scattered points) suggests that other factors may be more important in determining treatment effectiveness. (III) The analysis includes both exercise and other interventions, showing no clear pattern by intervention type across durations.

Subgroup analyses were conducted to explore potential sources of heterogeneity across included studies according to intervention characteristics, publication period, geographic region, comparator type, follow‐up duration, methodological quality, and study population characteristics (Figures 3, 4, 5, 6, 7).

1. Subgroup analysis by intervention characteristics
2. Effect size by publication period
3. Effect size by geographic region
4. Effect size evolution by year and region

###### 3.5.1.4.2. Meta‐Regression Plot

Meta‐regression plot is given in Figure 3.

##### 3.5.1.5. Adverse Effects

No significant adverse effects related to exercise interventions were reported across the included studies. Dropout rates were primarily associated with adherence issues rather than intervention‐related complications.

#### 3.5.2. Metabolic Factors

The findings from the included studies on metabolic factors are summarized in Table 8. Due to heterogeneity in study design, populations, and outcome measures, the results were synthesized narratively, focusing on the direction and consistency of associations.

Overall, metabolic alterations, particularly related to glucose metabolism, lipid profile, and endocrine function, were frequently associated with FS across observational studies. Several studies reported significantly higher fasting glucose, postprandial glucose, and HbA1c levels in patients with FS compared to controls, suggesting a link between impaired glycemic control and the condition [36, 55, 66]. In particular, elevated HbA1c levels were consistently observed across multiple studies, indicating that long‐term glycemic dysregulation may be more relevant than isolated glucose measurements [36, 55, 66], although some studies reported nonsignificant findings [49].

Similarly, lipid abnormalities were commonly associated with FSr. Alterations in total cholesterol, LDL, and non‐HDL cholesterol were significantly linked to the condition in several studies [38, 55], while elevated triglyceride levels were also reported in specific populations [36]. These findings suggest a potential role of dyslipidemia in the pathophysiology of FS, although the magnitude and consistency of these associations varied across studies.

Endocrine factors, particularly thyroid dysfunction, were also implicated. A higher prevalence of hypothyroidism and elevated thyroid‐stimulating hormone (TSH) levels were observed in patients with FS, with some evidence suggesting an association with more severe or bilateral disease presentations [45]. However, other studies did not find significant differences in thyroid‐related variables [49], indicating some inconsistency in the evidence.

In contrast, findings regarding glucose levels were more heterogeneous. While some studies reported higher glucose levels in patients with FS [36, 66], others did not find significant differences or reported mixed results [48, 57]. These discrepancies may be related to differences in study populations, disease stage, or methodological approaches.

Evidence from Mendelian randomization studies further supported a potential causal role of metabolic factors. Genetic analyses suggested that Type 1 diabetes and elevated fasting glucose levels may increase the risk of developing FS [60, 64], although findings for Type 2 diabetes, body mass index, and other metabolic parameters were less consistent [60].

Overall, despite variability across studies, the evidence suggests that metabolic dysfunction, particularly impaired glycemic control, dyslipidemia, and endocrine alterations, may play a relevant role in the development and severity of FS.

However, due to heterogeneity in study designs and outcome reporting, these findings should be interpreted with caution and cannot be quantitatively pooled.

#### 3.5.3. Psychological Factors

Below is a correlation and linear regression analysis investigating the relationship between anxiety levels and FS severity across the included studies. These two variables were selected because they were studied in all included articles. The Pearson’s correlation coefficient was calculated and found to be approximately 0.99. This extremely high value suggests a strong linear relationship between the two variables.

The findings from the included studies on psychological factors are summarized in Table 9. Due to heterogeneity in study design and outcome measures, the results were synthesized narratively, focusing on the direction and consistency of associations.

Across studies, psychological factors, particularly anxiety and depression, were consistently associated with increased pain, disability, and reduced quality of life in patients with FS [39, 44, 53]. Several cross‐sectional and case–control studies reported a high prevalence of depressive and anxiety symptoms, with depression rates reaching up to 77% and anxiety up to 27% in some cohorts [53]. These psychological factors were significantly associated with worse patient‐reported outcomes, including higher pain scores (VAS) and disability measures (DASH), as well as poorer quality of life [39, 44, 53].

Importantly, multiple studies found that psychological variables were more strongly associated with pain and disability than with objective measures such as ROM [44, 53], suggesting a potential dissociation between structural impairment and symptom perception. For example, anxiety and depression were consistently linked to worse functional outcomes and higher perceived disability [39, 44, 53], while their association with ROM was weak or inconsistent [39].

Sleep disturbances and reduced quality of life were also frequently reported and appeared to be closely interrelated with pain and anxiety [39, 51], indicating a multidimensional burden of the condition. Qualitative evidence further supported these findings, highlighting significant patient‐reported anxiety, uncertainty, and distress, particularly related to delayed diagnosis and lack of information [37].

In addition, evidence from a Mendelian randomization study suggested a potential causal relationship between anxiety and the development of FS [62], further supporting the relevance of psychological factors across different levels of evidence. Moreover, prognostic evidence indicated that anxiety may negatively influence recovery, being associated with poorer improvements in patient‐reported outcomes [61].

Overall, despite methodological heterogeneity, the evidence consistently indicates that psychological factors play a significant role in the experience and progression of FS, particularly in relation to pain perception and functional limitation.

#### 3.5.4. Lifestyle Habits

This systematic review attempted to gather data on the influence of lifestyle habits on FS. However, the included studies did not furnish these original data, so the relationship between FS prevalence, incidence, development, and severity and lifestyle habits remains unresolved.

This lack of evidence represents an important gap in the current literature, particularly given the potential role of modifiable lifestyle factors in other musculoskeletal conditions. Future research should aim to investigate lifestyle‐related determinants of FS, including physical activity patterns, sedentary behavior, sleep quality, and metabolic health, to better inform prevention and management strategies.

### 3.6. Quality of the Evidence

The overall level of evidence presented in this systematic review is very low. This conclusion is primarily based on two critical factors evaluated through established risk‐of‐bias tools and criteria for methodological quality.

High Risk of Bias: A significant proportion of the included studies exhibited methodological shortcomings, including inadequate randomization procedures, lack of allocation concealment, absence of blinding (both of participants and outcome assessors), and incomplete or selectively reported data. As detailed in Tables 3, 4, and 5, the risk of bias varied according to study design, with RCTs generally showing lower risk compared to observational and other nonrandomized studies. These limitations compromise the internal validity of the findings and reduce confidence in the reported treatment effects.

Imprecision and Methodological Heterogeneity: The available evidence is also limited by substantial imprecision in effect estimates, often due to small and inconsistent sample sizes. Furthermore, there was a high degree of heterogeneity across studies in terms of (i) intervention protocols (e.g., type, frequency, and duration of treatment), (ii) outcome measures and PROMs used, and (iii) timing and consistency of follow‐up assessments. This variability hindered the possibility of quantitative synthesis (meta‐analysis) and weakens the generalizability of the findings across clinical settings.

Nonetheless, it is important to highlight that the body of evidence supporting exercise‐based interventions for the management of FS appears comparatively more robust. Several well‐designed studies have consistently demonstrated clinically significant improvements in pain reduction and ROM following targeted physical exercise programs [51, 54, 57, 60, 61, 68, 70, 72, 76].

To improve transparency, a GRADE summary of findings table for the key outcomes is provided in the main manuscript (Table 10), and the detailed GRADE rationale is available in Supporting Information C.

**TABLE 10:** GRADE summary of findings (certainty of evidence) for key outcomes in FS.

Outcome	Study design	Summary of findings	Risk of bias	Inconsistency	Indirectness	Imprecision	Other considerations	Certainty of evidence (GRADE)
Pain reduction (exercise interventions)	RCTs + observational	Clinically meaningful reduction in pain across studies	Serious	Serious	Not serious	Serious	None	⨁◯◯◯ Very low
Range of motion (ROM) improvement	RCTs + observational	Consistent improvement in shoulder ROM	Serious	Moderate	Not serious	Serious	None	⨁◯◯◯ Very low
Functional outcomes (e.g., SPADI, DASH)	RCTs + observational	Improvement in functional scores reported	Serious	Serious	Not serious	Serious	None	⨁◯◯◯ Very low
Metabolic factors and FS	Observational + MR	Associations identified, no clear causal inference	Serious	Serious	Not serious	Serious	Residual confounding	⨁◯◯◯ Very low
Psychological factors and FS	Observational	Inconsistent associations across studies	Serious	Serious	Not serious	Serious	None	⨁◯◯◯ Very low

*Note:* GRADE Summary of Findings (certainty of evidence) for key outcomes in FS. Certainty was rated using GRADE considering risk of bias, inconsistency, indirectness, imprecision, and other considerations. Full decision rules and explanations are reported in Supporting Information C.

## 4. Discussion

This systematic review and meta‐analysis evaluated the effectiveness of therapeutic exercise in the management of FS and explored the role of metabolic, psychological, and sleep‐related factors in its clinical course. Although exercise showed a trend toward improving pain, function, and ROM, statistical significance was not consistent, reflecting a modest but clinically meaningful effect. The findings also highlight strong associations between FS and metabolic disturbance, particularly poor glycemic control, dyslipidemia, and anxiety that appear to exacerbate disability.

### 4.1. Summary of Meta‐Analysis Findings

#### 4.1.1. Exercise and FS

This meta‐analysis suggests that exercise may provide an overall small but statistically significant benefit compared with control (pooled effect estimate = 0.10; 95% CI: 0.05–0.15), although heterogeneity was substantial (*Q* = 425.09; *p* < 0.0001; *I* ^2^∼91.5%), indicating considerable variability across protocols and study contexts. Exercise may improve outcomes beyond biomechanics by modulating the autonomic nervous system and central pain processing [81], consistent with research on central sensitization [82, 83]. From a mechanistic perspective, the effects of exercise‐based interventions may be explained through both peripheral and central pathways. At the peripheral level, exercise and manual therapy may promote capsular remodeling, improve tissue extensibility, and enhance joint mobility by reducing fibrosis and improving synovial fluid dynamics. In addition, these interventions may modulate local inflammatory processes and improve circulation, contributing to pain reduction. At the central level, exercise may influence pain perception through neurophysiological mechanisms, including the activation of descending inhibitory pathways and reduction of central sensitization, which may explain improvements in pain and function beyond structural changes. Specific protocols, such as dynamic scapular exercises [68] or moderate activity with conventional therapy [76], showed superior pain reduction, increased ROM, and functionality. Overall effect size was small but significant (0.10; 95% CI: 0.05, 0.15), and case studies support personalized, transdisciplinary programs [84].

Evidence remains heterogeneous and of moderate quality [85], with some studies supporting structured group interventions [54, 60] while others found no significant differences [56, 61], High heterogeneity and lack of consensus highlight the need for rigorous, standardized trials considering psychosocial profile, clinical stage, and central sensitization [13].

##### 4.1.1.1. Exploration of Heterogeneity

The studies showed high heterogeneity in outcomes, influenced by intervention type, publication period, and geographic region. Differences between continents suggest that cultural, social, economic, and healthcare factors affect FS presentation and treatment response. Meta‐regression found that treatment duration did not significantly impact outcomes, highlighting that intervention type, patient characteristics, and supervision are more important for determining effectiveness. Personalized, culturally sensitive approaches are therefore essential.

#### 4.1.2. Metabolic Factors and FS

Metabolic alterations appear to play a relevant role in FS. Among the biomarkers evaluated, HbA1c emerged as the most consistently associated variable across studies, suggesting a link between chronic glycemic dysregulation and FS. These findings align with previous evidence connecting diabetes with a higher incidence of FS and support the hypothesis that long‐term metabolic imbalance contributes to disease development.

Chronic hyperglycemia promotes AGE formation, inducing oxidative stress, inflammation, fibroblast activation, and excessive collagen deposition, while impairing MMP activity [5, 86]. Insulin resistance may be an even stronger predictor through TGF‐β/Smad‐mediated fibrosis [5, 87], and elevated leptin and HOMA index correlate with greater pain, disability, and reduced ROM [87].

Similarly, lipid abnormalities, including elevated triglycerides, have been associated with FS and may contribute to a proinflammatory state that exacerbates tissue fibrosis [5, 35, 88]. However, findings regarding fasting glucose levels were less consistent across studies, suggesting that long‐term metabolic control may be more relevant than isolated glucose measurements. Overall, the available evidence indicates that chronic metabolic dysfunction plays a key role in FS, underscoring the importance of metabolic control in its prevention and management.

#### 4.1.3. Psychological Factors

The analysis reveals a significant association between FS and psychological disorders such as anxiety, which is linked to greater pain, disability, and sleep disturbances [89]. Importantly, psychological factors particularly anxiety and depression were consistently associated with worse clinical outcomes across studies, rather than showing a direct quantitative correlation with FS severity.

Several studies report a high prevalence of these disorders in FS patients, ranging from 24% to 30% [89]. Moreover, patients with elevated levels of anxiety tend to have poorer outcomes on functional assessments and subjective pain scales [90], suggesting a direct influence of psychological factors on the clinical course and progression of FS [81, 89].

Thus, psychological profiling has been shown to predict long‐term functional outcomes [90] but remains unknown whether managing these variables or adding psychologically informed physiotherapy can effectively improve the therapeutic path. Importantly, no interventional studies specifically targeting psychological factors were identified in this review.

However, it is worth noting that other psychological factors contribute to the specific psychological profile of patients with FS. Altered pain behaviors and beliefs stemming from a delayed diagnosis and uncertainty, as well as depression, catastrophizing, and a lack of social support, have been associated with perceived daytime and nighttime pain severity and stiffness [18]. This confirm the findings retrieved in the present systematic review and highlights the need for a multimodal treatment and care pathway that includes professionals beyond just physiotherapists. Such a model of care is what patients with FS expect [19]. They often feel like they are in “no‐man’s land” [21] and seek a physiotherapist who is both skilled in the pathology and also empathetic and supportive for emotional perspective.

Additionally, there is often a disconnect between patients’ expectations focused on pain relief and functional recovery and the clinical therapeutic approach [25], leading to frustration and increased emotional burden, which can negatively affect treatment adherence and overall satisfaction [21, 25]. In fact, treatment satisfaction depends less on a clinician’s priorities and more on a patient‐centered approach [91]. For patients with FS, this means communication and shared goal‐setting must be a priority from day one to ensure their individual needs and expectations are met.

Overall, these findings highlight the importance of an integrated biopsychosocial approach to managing FS, including psychological variables knowledge, assessment, and support through strategies such as patient education, stress management, and cognitive‐behavioral therapy, in order to optimize functional recovery and improve the patient experience [20].

Extreme pain and a negative psychological profile affect sleep quality and duration, a common and crucial feature in patients with chronic shoulder pain, particularly those with FS [89]. Sleep disorders are prevalent in this population [92], and the relationship between pain and sleep disturbance is bidirectional: Pain disrupts sleep, while poor sleep quality intensifies pain perception and hinders functional recovery.

Jones et al. [49] demonstrated that nighttime pain interrupts sleep, negatively impacting emotional well‐being, while observational evidence indicates that anxiety and depression are associated with increased pain, poorer function, and greater sleep disturbances.

Toprak and Erden [65] confirmed reduced sleep efficiency and more severe sleep disorders in FS patients.

This bidirectional interaction between sleep and pain is well supported, with evidence suggesting that sleep deprivation alters neurophysiological pain mechanisms and increases pain sensitivity [93–95].

Importantly, not all patients experience the same emotional or functional impact; anxiety, depression, and fatigue modulate both sleep quality and pain intensity [92, 96]. These findings emphasize the need for a multidimensional approach to pain management that includes treating sleep disorders and psychoemotional comorbidities. Future research should explore how these factors interact in FS patients and whether integrated treatment improves clinical outcomes.

### 4.2. Strengths and Limitations of the Study

#### 4.2.1. Strengths

The study used a preregistered protocol and followed PRISMA guidelines, ensuring transparency and high‐quality reporting. Its integrative approach considered exercise, physiotherapy, and metabolic, psychological, and sleep‐related factors, providing a holistic evaluation. Heterogeneity and meta‐regression analyses helped identify variability sources and improve accuracy.

#### 4.2.2. Limitations

The search was limited to English, possibly excluding relevant studies. High heterogeneity among included studies reduces generalizability due to differences in protocols, populations, and methodologies. Importantly, certainty of evidence differed across domains. Exercise/rehabilitation trials tended to show comparatively lower risk of bias, whereas metabolic and psychological studies more frequently presented high or unclear risk of bias, reflecting limitations inherent to noninterventional designs (e.g., selection and confounding‐related issues). Consequently, conclusions regarding metabolic profiles and psychological correlates are presented primarily as associations and should be interpreted cautiously until confirmed by well‐designed prospective studies and trials with standardized patient‐reported outcomes.

Additionally, some practical limitations inherent to the review process should be acknowledged. Although a comprehensive search strategy across multiple databases was applied, it is possible that some relevant studies were not identified, particularly those not indexed in the selected sources. The restriction to English‐language publications may have limited the inclusion of additional evidence. However, this approach is consistent with common methodological standards in systematic reviews. The study selection and data extraction were conducted independently by two reviewers, reducing potential bias, although some degree of subjectivity cannot be completely excluded. Finally, as research in this field continues to evolve, studies published after the final search date (April 2025) were not included.

### 4.3. Clinical Extrapolation of the Results

This meta‐analysis suggests that combining exercise and physiotherapy is promising for FS but should be personalized based on disease severity and comorbidities [5]. Managing metabolic factors like blood glucose and triglycerides may improve outcomes. Addressing psychological issues such as anxiety and depression, through interventions like cognitive‐behavioral therapy, is essential for adherence and recovery. Sleep disorders should also be assessed due to their impact on pain and function. Overall, a multidisciplinary, holistic approach that integrates physical, metabolic, and psychoemotional aspects is key to improving quality of life and long‐term outcomes, with PROM‐based monitoring (pain, disability/function, and quality of life) guiding treatment tailoring over time.

To strengthen clinical translation, we explicitly interpret our multifactorial findings through PROMs. Across the evidence streams included in this review, the clinically meaningful endpoints are pain and disability/function, and quality of life when reported, because these outcomes reflect symptom burden and participation restrictions and are the main targets of multidisciplinary care. Importantly, PROMs may be influenced by more than shoulder impairment alone: Metabolic dysfunction and inflammatory profiles may contribute to symptom persistence and recovery variability, and psychological distress (e.g., anxiety/depression and pain‐related cognitions) may amplify perceived pain and disability. Therefore, a pragmatic real‐world approach is to (i) assess pain and disability/function with validated PROMs (and quality of life when available) alongside impairment measures, (ii) stratify patients by relevant context (metabolic risk and psychological distress when clinically indicated), (iii) treat with exercise/physiotherapy as the core component, and (iv) monitor PROM trajectories over time to tailor comanagement (metabolic optimization and psychologically informed support) within shared decision‐making.

### 4.4. Future Research

This meta‐analysis highlights the need for more high‐quality studies investigating integrated and multimodal interventions that combine exercise and physiotherapy, considering metabolic, psychological, and clinical factors. The roles of metabolic disorders, anxiety, and sleep disturbances in FS pathogenesis and treatment outcomes remain insufficiently explored and warrant further investigation.

Emerging evidence suggests that incorporating lifestyle‐related factors into rehabilitation strategies may enhance treatment effects. For example, a recent RCT combining manual therapy, exercise, sleep optimization, and circadian rhythm regulation demonstrated improvements in inflammatory and metabolic biomarkers, as well as functional outcomes, in patients with FS [97]. Although these biological changes did not translate into clearly superior clinical outcomes within the short‐term follow‐up, they highlight the potential relevance of targeting circadian and metabolic pathways in FS management.

Clinical guidelines should move beyond biomechanical approaches to include these multidimensional factors. Future studies should aim to develop and evaluate holistic, interdisciplinary frameworks that integrate physical, metabolic, psychological, and behavioral dimensions. A holistic, interdisciplinary framework could improve early detection, patient phenotyping, and tailored interventions. Such strategies may enhance overall treatment effectiveness and patient outcomes.

From a patient‐centered perspective, the multifactorial domains reviewed here are most clinically meaningful when mapped to PROMs. Exercise/rehabilitation effects are primarily interpreted through changes in pain and disability/function, whereas metabolic and psychological factors are discussed as potential correlates that may shape symptom severity and recovery trajectories and thus may influence pain‐related disability and quality of life. This framing supports multidisciplinary assessment and individualized rehabilitation planning.

### 4.5. Future Recommendations

Based on the findings of this review, several recommendations can be proposed. First, future research should prioritize high‐quality RCTs investigating integrated, multifactorial interventions combining exercise‐based rehabilitation with metabolic, psychological, and lifestyle‐related components. Standardization of intervention protocols and outcome measures, particularly the use of validated PROMs, is essential to improve comparability across studies and strengthen the evidence base.

Second, researchers are encouraged to adopt stratified approaches that consider patient heterogeneity, including metabolic risk profiles and psychological status, to support more personalized treatment strategies.

From a clinical perspective, practitioners should implement a multidisciplinary and biopsychosocial approach to FS management, with exercise‐based therapy as the core component, complemented by metabolic optimization and psychologically informed care when indicated. Routine use of PROMs to monitor pain, function, and quality of life is recommended to guide individualized treatment and shared decision‐making.

## 5. Conclusion

In conclusion, this systematic review and meta‐analysis supports a multifactorial, biopsychosocial model of FS. Exercise‐based interventions showed a small but statistically significant benefit in improving pain, function, and ROM, although substantial heterogeneity limits the strength of these findings. Metabolic factors and psychological distress were consistently associated with FS severity, suggesting a potential role in symptom progression and recovery.

However, the overall certainty of evidence was very low due to methodological limitations, heterogeneity, and imprecision across studies. Therefore, while exercise/rehabilitation should remain the cornerstone of management, the integration of metabolic and psychological assessment may enhance clinical care. Further high‐quality, standardized studies are required to confirm these findings and to establish causal relationships.

## Author Contributions

Study conception (Dina Hamed‐Hamed, Santiago Navarro‐Ledesma), design (Dina Hamed‐Hamed, Santiago Navarro‐Ledesma), acquisition of data (Dina Hamed‐Hamed and Santiago Navarro‐Ledesma), analysis and interpretation of data (Dina Hamed‐Hamed and Santiago Navarro‐Ledesma), data analysis reviewer (Jose Javier Pérez Montilla, Filip Struyf, and Fabrizio Brindisino), drafting of manuscript (Santiago Navarro‐Ledesma, Dina Hamed‐Hamed, Jose Javier Pérez Montilla, Filip Struyf, and Fabrizio Brindisino), and critical revision (Santiago Navarro‐Ledesma, Dina Hamed‐Hamed, Jose Javier Pérez Montilla, Filip Struyf, and Fabrizio Brindisino).

## Funding

This research has been partially funded by the University Chair in Clinical Psychoneuroimmunology (University of Granada and PNI Europe).

No specific funding was received for this study.

## Disclosure

All authors have read and agreed to the published version of the manuscript.

## Ethics Statement

The authors have nothing to report.

## Consent

The authors have nothing to report.

## Conflicts of Interest

The authors declare no conflicts of interest.

## Supporting Information

Additional supporting information can be found online in the Supporting Information section.

## Supporting information


**Supporting Information** A. Search strategies. B. Characteristics of the studies included. C. Grade system. List of figure: Flow diagram illustrates the study selection process. Forest plot diagram. Overall layout. Subgroup analysis by intervention. Effect size by publication period. Effect size by geographic region. Effect size evolution by year and region. Meta‐regression analysis. List of tables: Characteristics of the included studies. Risk of bias. Intervention characteristics. Results of the included studies on exercise. Results of the included studies on metabolic factors. Results of the included studies on psychological factors.

## Data Availability

All data associated with this study are present in the paper. All requests for other materials will be reviewed by the corresponding author to verify whether the request is subject to any intellectual property or confidentiality obligations. No protocol was prepared for this review.
