# Supplementary material for: Rehabilitation of Frozen Shoulder: A Systematic Review and Meta‐Analysis of Multifactorial Interventions
Source: Pain Res Manag. 2026 Jun 17;2026:3820129. doi: 10.1155/prm/3820129 (PMC13273838; doi:10.1155/prm/3820129)
Supplement: Supplementary file 1 — Supporting Information A. Search strategies. B. Characteristics of the studies included. C. Grade system. List of figure: Flow diagram illustrates the study selection process. Forest plot diagram. Overall layout. Subgroup analysis by intervention. Effect size by publication period. Effect size by geographic region. Effect size evolution by year and region. Meta‐regression analysis. List of tables: Characteristics of the included studies. Risk of bias. Intervention characteristics. Results of the included studies on exercise. Results of the included studies on metabolic factors. Results of the included studies on psychological factors. [file PRM-2026-3820129-s001.docx]

**Supplementary material**

**Supplementary material A: Search Strategies**

| Database | Search Strategies |
| --- | --- |
| LILACS E IBECS | Tw:((“Adhesive capsulitis” OR “Frozen shoulder”) AND ("Circadian Clocks" OR "Circadian Rhythm" OR “Chronobiology Discipline" OR "Sleep Wake Disorders" OR "insulin" OR "Fasting" OR "Caloric Restriction" OR "Diet, Ketogenic" OR “Nutritional strategies” OR “Therapy”) AND ("Pain" OR "Chronic Pain" OR "Healthy" OR "Healthy Lifestyle" OR “Quality of life” OR "Quality of Life/psychology" OR “Psychological factors” OR “Funcionality” )) OR ((“Frozen shoulder”) AND mh:("Circadian Clocks" OR "Circadian Rhythm" OR "Chronobiology Discipline" OR "Sleep Wake Disorders" OR "insulin" OR "Fasting" OR "Caloric Restriction" OR "Diet, Ketogenic") AND ("Pain" OR "Chronic Pain" OR "Healthy Lifestyle" OR "Quality of Life/psychology")) |
| EBSCO | ((“Adhesive capsulitis” OR “Frozen shoulder”) AND ("Circadian Clocks" OR "Circadian Rhythm" OR “Chronobiology Discipline" OR "Sleep Wake Disorders" OR "insulin" OR "Fasting" OR "Caloric Restriction" OR "Diet, Ketogenic" OR “Nutritional strategies” OR “Therapy”) AND ("Pain" OR "Chronic Pain" OR "Healthy" OR "Healthy Lifestyle" OR “Quality of life” OR "Quality of Life/psychology" OR “Psychological factors” OR “Funcionality” )) OR (("Frozen shoulder") AND MH: (("Circadian Clocks" OR "Circadian Rhythm" OR "Chronobiology Discipline" OR "Sleep Wake Disorders" OR "insulin" OR "Fasting" OR "Caloric Restriction" OR "Diet, Ketogenic") AND ("Pain" OR "Chronic Pain" OR "Healthy Lifestyle" OR "Quality of Life/psychology"))) |
| Web Of Science | TS=((“Adhesive capsulitis” OR “Frozen shoulder”) AND ("Circadian Clocks" OR "Circadian Rhythm" OR “Chronobiology Discipline" OR "Sleep Wake Disorders" OR "insulin" OR "Fasting" OR "Caloric Restriction" OR "Diet, Ketogenic" OR “Nutritional strategies” OR “Therapy”) AND ("Pain" OR "Chronic Pain" OR "Healthy" OR "Healthy Lifestyle" OR “Quality of life” OR "Quality of Life/psychology" OR “Psychological factors” OR “Funcionality”) |
| Scopuss | ((“Adhesive capsulitis” OR “Frozen shoulder”) AND ("Circadian Clocks" OR "Circadian Rhythm" OR “Chronobiology Discipline" OR "Sleep Wake Disorders" OR "insulin" OR "Fasting" OR "Caloric Restriction" OR "Diet, Ketogenic" OR “Nutritional strategies” OR “Therapy”) AND ("Pain" OR "Chronic Pain" OR "Healthy" OR "Healthy Lifestyle" OR “Quality of life” OR "Quality of Life/psychology" OR “Psychological factors” OR “Funcionality” )) OR (("Frozen shoulder") AND ("Circadian Clocks" OR "Circadian Rhythm" OR "Chronobiology Discipline" OR "Sleep Wake Disorders" OR "insulin" OR "Fasting" OR "Caloric Restriction" OR "Diet, Ketogenic") AND ("Pain" OR "Chronic Pain" OR "Healthy Lifestyle" OR "Quality of Life/psychology")) |

**Supplementary material B: characteristics of the studies included**

| **Author** | **Design / Sample** | **Focus Area** | **Intervention / Analysis** | **Main Outcomes** |
| --- | --- | --- | --- | --- |
| Salek et al.31 | Case-control | Metabolic | Fasting/postprandial glucose, HbA1c, triglycerides | Metabolic profile |
| Jones et al.32 | Qualitative, n=12 | Psychological | Patient experiences and priorities in different FS phases | Subjective perceptions |
| Sung et al.33 | Case-control, n=300/900 | Metabolic | Lipid profile (TC, LDL, HDL, TG) | Metabolic dysfunction |
| Ding et al.34 | Cross-sectional | Psychological | HADS-A/D, VAS, ROM, SPADI, SST | Anxiety, depression, pain |
| Russel et al.35 | RCT, 3 groups, n.s. | Physical | Group exercise vs. individualized physiotherapy vs. home exercise | Pain, mobility |
| Gutierrez et al.36 | RCT, n=57 | Physical | Posterior mobilization + cycling vs. conventional physiotherapy | ROM, pain, Constant |
| Ali et al.37 | RCT, n=44 | Physical | Maitland mobilization + exercise vs. exercise only | VAS, ROM, SPADI |
| Celik et al.38 | RCT, n=30 | Physical | Mobilization + stretching vs. stretching only | DASH, Constant |
| Bagheri et al.39 | Questionnaire-based | Psychological | VAS, SF-36, DASH, Hamilton A/D | Pain, QoL, disability |
| Schiefer et al.40 | Case-control | Metabolic | Prevalence of hypothyroidism (standardized criteria) | Thyroid dysfunction |
| Horst et al.41 | Double-blind RCT, n=66 | Physical | Structural vs. activity-oriented physiotherapy | Pain, function |
| Robinson et al.42 | RCT, n=41 | Physical | Supervised physio + home exercise vs. home only | OSS, ROM, VAS, EQ-5D |
| Safran et al.43 | Case series | Metabolic | Fasting glucose levels | Glucose imbalance |
| Chan et al.44 | Retrospective, n=24,417 | Metabolic | Cumulative HbA1c, logistic regression | Diabetes-FS link |
| Elhamed et al.45 | RCT, n=30 diabetics | Physical | Lower trapezius strengthening vs. traditional PT | Scapular tilt |
| Toprak et al.46 | Comparative | Psychological | BAI, BDI, PSQI, WHOQoL-BREF, VAS | Anxiety, sleep, QoL |
| Rai et al.47 | Comparative | Metabolic | OGTT results in FS patients | Diabetes/prediabetes prevalence |
| Ebrahimzadeh et al.48 | Cross-sectional, n=120 | Psychological | Depression, anxiety vs. ROM, pain | Psychological impact |
| Mohamed et al.49 | RCT, n.s. | Physical | Scapular recognition exercises | Scapular rotation, ROM |
| Park et al.50 | Case-control, n=151/453 | Metabolic | Glucose, HbA1c, lipids, CRP-hs | Inflammation, metabolism |
| Lin et al.51 | Pilot study, n=48 | Physical | PNF vs. manual therapy | CHL/CAR thickness, VAS, ROM |
| Jessic et al.52 | Prospective, n=26 | Metabolic | HbA1c, ROM, time-to-treatment | Functional progression |
| Razzaq et al.53 | Single-blind RCT, n=64 | Physical | MWM vs. MET + conventional PT | VAS, ROM, SPADI |
| Wang et al. | RCT /n=40 | Physical exercie | \|  \| \| --- \|  \| Neuromuscular exercises (NME) vs. strengthening exercises + physiotherapy over 8 weeks \| \| --- \| | Both groups improved in pain and AROM; group-time interaction analyzed |
| Yang et al.55 | Mendelian radomization study | Metabolic | Not applica | Not applica |
| Romeo et al.56 | Retrospective cohort | Psychological | PROMIS-UE, VAS, P-UE over 1 year | Patient-reported outcomes |
| Ouyang et al.57 | Mendelian Randomization | Psychological | Genetic link: anxiety ↔ FS | MR analysis (IVW, Egger) |
| Sheikh et al.58 | RCT, n=44 diabetics | Physical | Moderate physical activity + PT vs. PT only | DASH, NPRS, ROM |
| Chen et al.59 | Mendelian Randomization | Metabolic | Genetic link: diabetes ↔ FS | MR causal inference |
| Hamed et al.60 | Cross-sectional, n=32 | Metabolic | Metabolites, pain, liver-thyroid interaction | Functional-metabolic link |
| Mertens et al.61 | Cross-sectional, n=35 FS / 35 controls | Metabolic | Finger-prick glucose, HbA1c (A1CNow+) | Glycemic status |

Abbreviations: RCT = randomized controlled clinical trial;

**Supplementary material C: GRADE SYSTEM.**

Author(s): Dina Hamed-Hamed, Jose Javier Pérez Montilla, Filip Struyf, Santiago Navarro-Ledesma.

Question: Physical therapy techniques compared to life style for FS

Setting: Department of Physical therapy, University of Granada, Spain Faculty of Health Sciences, Campus of Melilla

**Supplementary Table 2: GRADE System**

**CI:** confidence interval

**Explanations**

a. Most of the biases had an unclear risk, as no intervention was performed.

b. In relation to the studies that applied exercise, it is observed that Mulligan's mobilization with movement, combined with conventional physiotherapy treatment, generates significantly greater improvements in pain reduction, increased range of motion, and optimization of functional activity. Additionally, there is a relationship between anxiety and frozen shoulder, as well as an association with various metabolic factors such as diabetes.
